# Microbial synthesis of short-chain sophorolipid esters using yeasts of the *Starmerella* clade

**DOI:** 10.1186/s13568-026-02130-5

**Published:** 2026-10-24

**Authors:** Eugen Banmann, Enes Medet Aksoy, Ulrich Schörken

**Affiliations:** TH Köln – Campus Leverkusen, Campusplatz 1, 51379 Leverkusen, Germany

**Keywords:** Biosurfactants, *S. bombicola*, *Starmerella* clade, Sophorolipids, Sophorolipid esters, Alcohol feeding

## Abstract

Acetylated sophorolipid (SL) esters exhibit surface-active as well as bioactive properties. Their synthesis requires a three-step route comprising microbial production of the SL lactone followed by chemical transesterification and enzymatic re-acetylation. Here, a direct microbial synthesis of SL esters was investigated using three-component feeding of glucose, oleic acid and a wide variety of alcohols. Straight-chain primary alcohols with chain lengths of C3–C12 impaired SL production of *Starmerella bombicola* severely, whereas toxicity of short-chain primary, secondary and branched-chain alcohols was significantly lower, and total SL production of up to 60 g l^−1^ was obtained in initial shake-flask cultures. Besides lactonic, acidic and bolaform SLs, acetylated SL esters were detected in small quantities in the fermentation broth. Upon continuous alcohol feeding the SL ester fraction could be increased to 10–40 g l^−1^ of methyl, ethyl, isopropyl and isobutyl SL esters under optimized conditions. Following chromatographic purification, the ester structures were verified by LC–MS and NMR spectroscopy. The synthesis of the isopropyl SL ester was scaled up successfully to a 2 l bioreactor, yielding 100 g l^−1^ total SL including 30 g l^−1^ of the isopropyl ester. To elucidate the SL ester formation capabilities within the *Starmerella* clade, five additional species were screened. SL synthesis was shown for the first time with *S. caucasica* and *S. davenportii*. With all strains, including ω-hydroxylating open-chain SL producing *S. kuoi* and *S. batistae*, SL ester formation was detected. Thus, the ability for SL ester synthesis seems to be ubiquitous among SL producing *Starmerella* strains.

**Supplementary Information:**

The online version contains supplementary material available at https://doi.org/10.1186/s13568-026-02130-5.

## Introduction

**Fig. 1:**
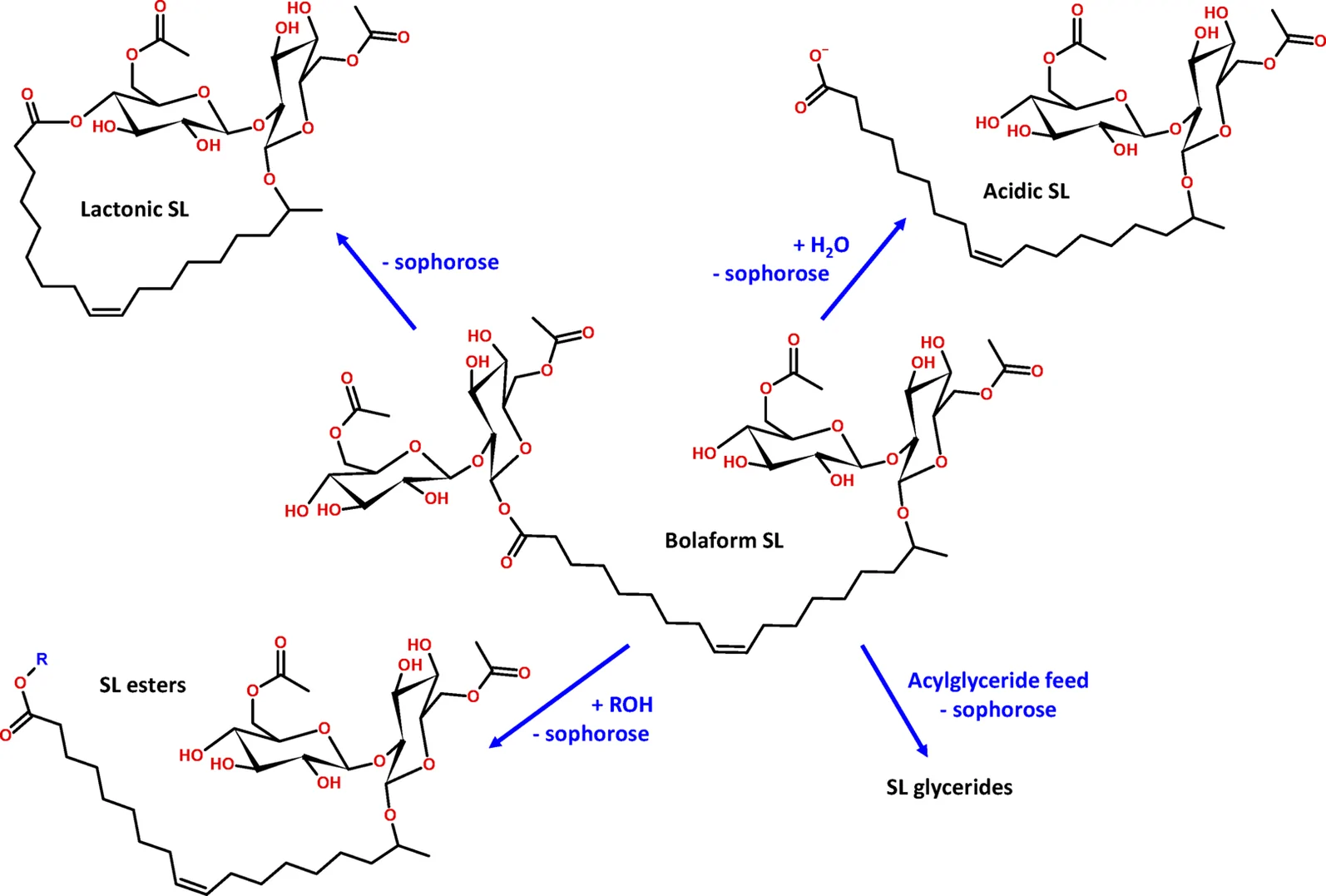
Principal outline of SL lactone (upper left), acid form (upper right) and glycerol ester (lower right) formation catalyzed by lactone transesterase SBLE as recently described (Roelants et al. 2024; Bovijn et al. 2024) and potential route to SL esters via alcohol feeding (lower left, exemplary ester structures are provided in Fig. S1)

Biobased glycolipids are gaining importance in the chemical and detergent industry. Besides long-known chemically manufactured alkyl polyglycosides, microbial rhamno- and sophorolipids (SLs) entered the market for cleaning and detergent applications (Schörken et al. 2017). Several manufacturers worldwide, including Evonik, Amphistar and Holiferm in Europe (Wongsirichot and Winterburn 2025), produce SLs on an industrial scale. Tremendous progress has been made in recent years in production efficiency, for example through in situ product removal (Dierickx et al. 2022; Wongsirichot and Winterburn 2025), and regarding sustainability, through the use of waste materials for production (Hu et al. 2021). The main advantages of SL production are high production titers achieved with the natural producer *Starmerella bombicola* and low foaming of the lactonic fermentation product, suitable for hard-surface cleaning. Upon chemical ring opening and deacetylation, a higher-foaming product for body care products is obtained (Bollmann et al. 2019). To increase the structural variety of SLs for a broader application profile, several approaches have been pursued. Chemical modification enables access to a variety of SL derivatives, summarized in recent reviews (Delbeke et al. 2016; Pala et al. 2023). Another approach is biological tailoring of SLs by strain selection, substrate variation and genetic engineering of the SL pathway (Li et al. 2021; Kobayashi and Fukuoka 2025). In this respect, the SL biosynthetic pathway had to be revised. A novel bolaform SL was identified as an intermediate (Fig. 1), which serves as the precursor for the formation of lactonic and acidic SLs catalyzed by lactone transesterase SBLE (Diao et al. 2024; Roelants et al. 2024). In a recent patent application, it was shown that SBLE is also capable of catalyzing an interesterification reaction of the bolaform SL with acylglycerides leading to SL glycerides (Bovijn et al. 2024). SL glycerides were initially found as trace compounds in fermentations with triglyceride oils as lipid substrates (Nuñez et al. 2001) and several SL glyceride esters have since been isolated and structurally characterized (Kobayashi et al. 2023, 2024). In these compounds, the SL carboxyl group is attached to glycerol and mono-, di- and triesters of SL have been detected.

In contrast to SL glyceride formation from triglyceride oils, the corresponding formation of SL alkyl esters by feeding fatty acid esters proved to be difficult. When methyl esters like biodiesel or methyl erucate were used as substrates, some SL methyl esters were produced; however, the majority of the SL fraction consisted of lactonic and acidic SLs with a composition shift towards the acidic open-chain species (Ashby et al. 2005; Shin et al. 2010; Kim et al. 2020). This effect was used to optimize acidic SL production to > 100 g l^−1^ using *Starmerella floricola* with methyl ester and waste glycerol as substrates (Konishi et al. 2018). In a comparative analysis, methyl, ethyl and propyl esters of soybean oil were used as substrates for SL production. In all cases, a high proportion of acidic SLs was observed, and in the case of the ethyl and propyl esters, no SL ester could be detected (Ashby et al. 2006). Thus, it seems that SL alkyl esters with ethanol or longer-chain alcohols are not accessible by direct feeding of the corresponding fatty acid esters.

An alternative approach is the chemical or chemo-enzymatic modification of SLs. Alkaline or acid-catalyzed transesterification enables the synthesis of non-acetylated SL esters with varying alcohol chain length. For the SL methyl ester, an improved oil-removal capacity from contaminated soil was recently reported in comparison to the SL lactone (Lei et al. 2025). Upon re-acetylation of the sophorose primary hydroxyl groups with immobilized *Pseudozyma aphidis* (formerly *Candida antarctica*) lipase B and vinyl acetate as acyl donor, the diacetylated SL esters became accessible (Bisht et al. 1999; Koh et al. 2017). Both the diacetylated and non-acetylated esters exhibited interesting surface-active properties, which differed significantly depending on hydrophobic tail length and degree of sophorose acetylation (Koh et al. 2017). These SL esters, including the diacetylated ethyl ester, were shown to possess anticancer activity against human pancreatic and breast cancer cells (Miceli et al. 2022; Roberge et al. 2023). Additionally, antimicrobial activity of the diacetylated SL ethyl ester against *Bacillus cereus* was reported (Totsingan et al. 2021).

In early studies by Tulloch and Spencer, the formation of an SL ester with a long-chain C36 tail was observed with oleyl alcohol as substrate (Tulloch and Spencer 1972). This formation can be explained by partial oxidation of the alcohol into 17-hydroxyoleic acid for SL formation and subsequent transesterification with residual oleyl alcohol. The formation of the long-chain esters may follow the route via the bolaform SL as depicted in Fig. 1. Based on this finding and the assumption that oxidation of long-chain alcohols would be low in *S. bombicola* in the presence of fatty acids due to downregulation of alkane assimilation (Hommel and Ratledge 1990; van Bogaert et al. 2009), we previously investigated the mixed feeding of fatty acids and long-chain alcohols. Long-chain SL esters could be tailored by varying the fatty acid and alcohol feed, and products with good emulsification potential were obtained in high yields (Zerhusen et al. 2020). Given the attractive surfactant properties and biological activities of short-chain SL esters, a direct microbial synthesis would be a significant advantage over the current synthesis approaches. However, SL ester share decreased rapidly when alcohols of a chain length below C14 were fed to *S. bombicola* (Zerhusen et al. 2020) and this prompted us to investigate whether suitable three-component feeding strategies enable a direct access to acetylated SL esters with short-chain alcohols. Besides analysis of the alcohol substrate spectrum and their inhibitory effects, we established feeding as a tool for process optimization and determined whether SL alkyl ester formation is a general trait of the *Starmerella* clade. For this, we compared six different *Starmerella* species with two of them, *S. davenportii* and *S. caucasica*, not verified as SL producers before.

## Materials and methods

### Materials and microorganisms

Oleic acid (composition C18:1, 91.6%; C16:0, 3.5%; C18:0, 2.0%; C18:2, 1.6%; C22:0, 0.5%; C20:1, 0.3%; C20:0, 0.2%; C16:1, 0.2%; C24:0, 0.2%) was kindly provided by DAKO AG (Wiesentheid, Germany). The enzymatic assay kit ENZYTEC ᴅ-glucose (E1210) was purchased from R-Biopharm AG (Darmstadt, Germany). All other reagents and solvents were purchased from Carl Roth GmbH (Karlsruhe, Germany), Sigma-Aldrich (St. Louis, MO, USA), and Fisher Scientific GmbH (Schwerte, Germany). The following alcohols with abbreviations given in parentheses were used in the feeding studies: (primary straight-chain alcohols) methanol (C1), ethanol (C2), propanol (C3), butanol (C4), hexanol (C6), octanol (C8), decanol (C10), dodecanol (C12), tetradecanol (C14), hexadecanol (C16), octadecanol (C18), oleyl alcohol (C18:1); (secondary alcohols) isopropanol (2s-C3), 2-butanol (2s-C4), 2-pentanol (2s-C5), 3-pentanol (3s-C5); (tertiary alcohols) *tert*-butanol (t-C4), *tert*-pentanol (t-C5); (branched-chain alcohols) isobutanol (2i-C4), 2-methyl-1-butanol (2i-C5), 3-methyl-1-butanol (3i-C5) and 2-ethyl-1-hexanol (2i-C8). The structures of all alcohols are shown in Fig. S2. Sophorolipid production was investigated using *Starmerella bombicola* Y-17069, *S. riodocensis* Y-27859 and *S. kuoi* Y-27208, which were obtained from the ARS Culture Collection of the U.S. Department of Agriculture (Peoria, IL, USA). In addition, *S. batistae* CBS 8550, *S.* *caucasica* CBS 12650 and *S. davenportii* CBS 9069 were obtained from the Westerdijk Fungal Biodiversity Institute (Utrecht, the Netherlands). All strains were maintained as glycerol stocks at −80 °C.

### Microbial synthesis of SL esters in shake-flask cultures

A preculture of *S. bombicola* was prepared by inoculating cells from an agar plate into 100 ml of YM medium (20 g l^−1^ glucose, 5 g l^−1^ peptone, 3 g l^−1^ yeast extract, and 3 g l^−1^ malt extract) and culturing for 24 h at 30 °C in a rotary shaker at 140 rpm. The SL production medium contained 100 g l^−1^ glucose, 5 g l^−1^ citric acid, 1 g l^−1^ or 3 g l^−1^ yeast extract, 1 g l^−1^ MgSO_4_, and 0.1 g l^−1^ NaCl and was adjusted to pH 4.5 with NaOH. The cells of the preculture were centrifuged (2000 × *g*, 5 min), washed with the production medium and centrifuged again before inoculation of 10 ml of production medium to an initial OD_600_ of 1 in 250 ml baffled shake-flasks. Oleic acid (10% v/v) was added directly to the flask at the start of cultivation. Cultivation was performed at 140 rpm and 25 °C for 14–21 days. Glucose concentration was analyzed periodically, and glucose solution (200 g l^−1^) was added to maintain a glucose concentration of ≥50 g l^−1^ in the culture medium. In detail, the broth was adjusted to a glucose concentration of 70 g l^−1^ when its level fell below 50 g l^−1^ and this was typically required up to 3 times per cultivation. Samples were taken every 24 h and analyzed directly by HPLC-ELSD for SL production. At the start of SL production, alcohol substrates were added to the culture and the formation of SL species was monitored.

Alcohol type, alcohol concentration and feeding regime were varied in the screening experiments and are described in detail in the results section. SL ester synthesis with the strains *S. batistae*, *S. caucasica*, *S. davenportii*, *S. riodocensis* and *S. kuoi* was performed under the same conditions and all experiments were conducted at least in duplicate.

### Production of SL isopropylester in 2 l scale

Bioreactor cultivation was performed in a 2.5 l benchtop bioreactor (Bioengineering AG, Wald, Switzerland) using *S. bombicola*. Precultures were prepared as described for shake-flask cultivations, and the entire biomass was washed and transferred to the bioreactor at inoculation. The production medium (2 l) was composed of 100 g l^−1^ glucose, 6 g l^−1^ yeast extract, 5 g l^−1^ citric acid, 1 g l^−1^ MgSO_4_, and 0.1 g l^−1^ NaCl, and was adjusted to pH 4.5 with NaOH. During fermentation, the pH was maintained above 3.5 by the addition of 1 M NaOH. Oleic acid (10% v/v) was added directly to the fermenter before sterilization. Fermentation was carried out at 25 °C for 14 days with an aeration rate of 2 vvm. The stirring rate was varied between 1000 and 1400 rpm to maintain dissolved oxygen above 50% air saturation. The glucose concentration was adjusted during fermentation as described for the shake-flask experiments. Isopropanol was added at a rate of 200 mM per day after the onset of SL production.

### Determination of glucose concentration

Glucose concentrations in the culture broth were determined enzymatically using the ENZYTEC ᴅ-glucose assay (E1210, R-Biopharm AG, Darmstadt, Germany). The assay is based on the phosphorylation of ᴅ-glucose to glucose−6-phosphate by hexokinase in the presence of ATP, followed by oxidation of glucose−6-phosphate to gluconate−6-phosphate by glucose−6-phosphate dehydrogenase. The NADPH formed is stoichiometrically equivalent to the amount of ᴅ-glucose present and was quantified photometrically at 340 nm. Prior to analysis, culture samples were centrifuged (3300 × *g*, 15 min) and the cell-free supernatant was diluted with deionized water to bring the glucose concentration within the linear range of the assay. Measurements were carried out according to the manufacturer's protocol.

### SL extraction and purification

SLs were extracted from cell cultures by adding an equal volume of ethyl acetate. For shake-flask cultures, the entire culture was used for SL extraction, whereas for bioreactor cultivations, the heavy SL phase was withdrawn through the bottom outlet of the fermenter prior to extraction. Phase separation was performed using a separating funnel, and extraction was repeated twice. The combined ethyl acetate phases were washed with an equal volume of water, dried over anhydrous Na_2_SO_4_, filtered, and evaporated under reduced pressure at 40 °C using a rotary evaporator.

Individual SL congeners were purified by flash chromatography on a Büchi Pure C-815 system equipped with UV and ELSD detectors, using a FlashPure Select C18 column (40 g, 30 µm, spherical). For each extract, 2 g was dissolved in acetonitrile/water/formic acid (50:50:0.1, v/v/v) to reduce viscosity, and the resulting solution was loaded onto the column. Elution was carried out at a flow rate of 30 ml min^−1^ using a linear gradient of water and acetonitrile, each containing 0.1% (v/v) formic acid, from 50:50 to 10:90 (v/v) over 40 min. Fractions containing the target compounds were combined and concentrated under reduced pressure, and the residues were crystallized from acetone/hexane and dried under vacuum to afford the products as crystalline solids. Purified compounds were used for HPLC-ELSD detector calibration and NMR structure elucidation.

### HPLC-ELSD analysis

Sampling of the cultures was performed by removing 100 μl of culture and diluting 1:60 in an acetonitrile/water/formic acid mixture (80:20:0.1). After vigorous vortexing, the samples were centrifuged (3300 × *g*, 15 min) for cell removal and applied directly for LC analysis. The samples were analyzed by HPLC using a Nexera XR system (Shimadzu Corp., Japan) equipped with an ELSD 100 detector (VWR Collection, United States) used with nitrogen at 2.5 bar and 50 °C for nebulization. The equipment was controlled by LabSolutions software (version 5.132) from Shimadzu. A LaChrom II C18 reverse-phase column (4.6 mm × 250 mm, 5 μm, Hitachi Ltd. Corp., Japan) was used at a constant flow rate of 1 ml min^−1^ at 50 °C with a water–acetonitrile (ACN) gradient supplemented with 0.1% (v/v) formic acid. Calibration of the ELSD detector was performed with purified SL species as outlined in Figs. S3 and S4.

Method A was used for routine analysis and elution conditions were as follows: start at 50% ACN, 1.5 min to 70% ACN, 1.5 min to 80% ACN, 12 min to 90% ACN and regeneration to 50% ACN. For hydrophobic esters (> C30), a prolonged gradient was used starting from 50% ACN, 1.5 min to 70% ACN, 1.5 min to 80% ACN, 2 min to 90% ACN, 15 min to 99% ACN and regeneration to 50% ACN. For C36 ester analysis, 99% ACN was kept for an additional 10 min. For detailed structural separation of SL congeners, method B was applied at 30 °C with elution conditions as follows: start at 50% ACN, 60 min to 90% ACN, hold at 90% ACN for 30 min and regeneration to 50% ACN. The injection volume for all methods was set to 10 μl, but reduced to 5 or 2 μl in case the SL concentration exceeded the upper limit of the linear detection range.

### HPLC–MS analysis

Mass spectrometric analyses were performed using a Shimadzu LC-30AD with an SPD M20A diode array detector and an LCMS-2020 single quadrupole mass spectrometer with atmospheric pressure chemical ionization (APCI-MS) equipped with an Orbit 100 C18 (50 × 4.6 mm, 5 µm) column. The mass spectrometer was run in positive and negative ion modes in the *m*/*z* range of 50–1600 at 15,000 u s^−1^ and an event time of 0.15 s. Corona needle voltage was set to 4.5 kV. A gradient of water and ACN, each supplemented with 0.1% (v/v) formic acid, was applied at a constant flow rate of 1 ml min^−1^ and a column temperature of 50 °C. The elution conditions were as follows: initial concentration of 20% ACN, linear gradient to 90% ACN in 4 min, hold 90% ACN for 2 min and regeneration to 20% ACN. Sample injection volume was set to 5 μl.

### NMR spectroscopy

NMR spectra were recorded with a 400 MHz Bruker Ascend 400 in deuterated dimethyl sulfoxide (DMSO-d6). Besides ^1^H and ^13^C NMR analysis, 2D-NMR experiments were used for structural analysis. Homonuclear bond correlation was elucidated with COSY experiments, heteronuclear bond correlation with HSQC and HMBC experiments. The software MestReNova 14.3.3 (Mestrelab Research S.L.) was used for data analysis.

### Bioinformatics analysis

Sequence analysis of homologs to *S. bombicola* transesterase SBLE was done by NCBI BLAST search (https://blast.ncbi.nlm.nih.gov/blast/Blast.cgi) (Altschul et al. 1990) of the genome sequences of *S. riodocensis* NRRL Y-27859, *S. kuoi* NRRL Y-27208*, S. batistae* SB001*, S. davenportii* CBS 9069 and *S. stellata* NRRL Y-1446 and nucleotide sequences of the closest matching sequences were translated with the Expasy Translate tool (https://web.expasy.org/translate/). Pairwise protein sequence alignments were done with EMBOSS Needle (https://www.ebi.ac.uk/jdispatcher/psa/emboss_needle) and multiple sequence analysis and phylogenetic analysis with Clustal Omega (https://www.ebi.ac.uk/jdispatcher/msa/clustalo) using the EMBL-EBI server (Madeira et al. 2024).

## Results

### SL ester synthesis with primary alcohols: toxicity analysis and structural elucidation

In our previous publication, we investigated the effect of fatty alcohol supplementation on SL composition and demonstrated that *S. bombicola* is capable of synthesizing long-chain SL esters (Zerhusen et al. 2020). However, reducing the alcohol chain length from C18 to C12 or C10 significantly decreased overall SL production and SL ester formation. To elucidate SL synthesis and alcohol toxicity as a function of chain length, we conducted shake-flask cultivations with primary alcohols ranging from C1 (methanol) to C18 (oleyl and stearyl alcohol), which were added in a concentration of 150 mM each. Since alcohol dosing was started at the onset of SL production, the term toxicity describes the impairment of metabolic activity of the resting SL-producing cells. Monitoring of individual SL species was done by direct dilution of homogenized culture samples in an acetonitrile–water-formic acid mixture to enable simultaneous analysis of all SL species in the broth by subsequent LC-ELSD and -MS analysis.

**Fig. 2:**
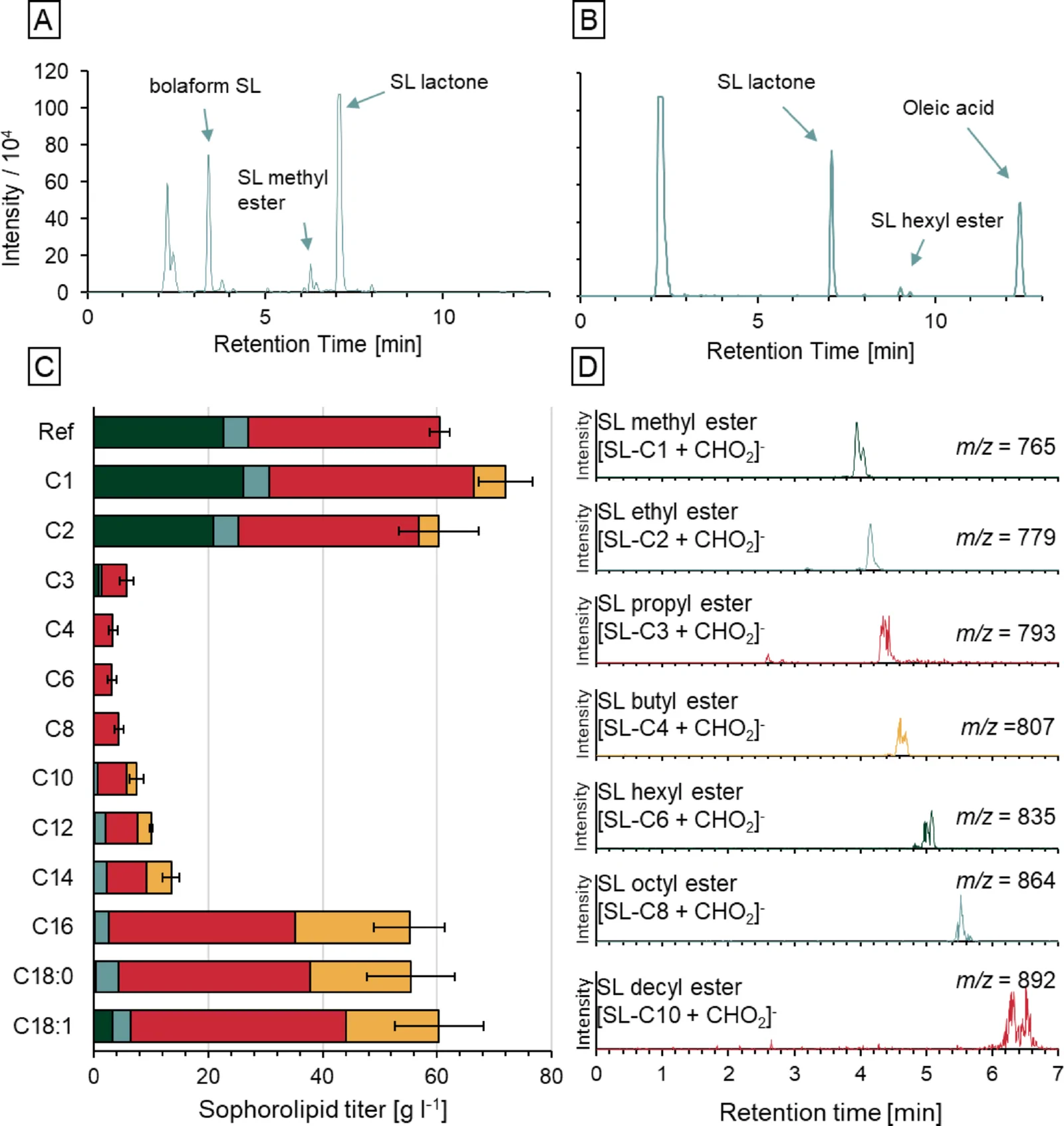
HPLC-ELSD chromatograms of *S. bombicola* culture broths exemplarily shown for supplementation with methanol (**A**) and hexanol (**B**) in the presence of 3 g l^−1^ yeast extract; **C** SL titers and species analysis of cultivations in the presence of 1 g l^−1^ yeast extract and 150 mM of alcohols ranging from C1 (methanol) to C18:1 (oleyl alcohol) in comparison to reference cultivation without alcohol; green, cyan, red and yellow bars represent bolaform, open chain, lactonic and esterified SL respectively; **D** Overlay of extracted ion chromatograms (EICs) for SL ester [M+CHO_2_]^−^ ions (*m/z* values for diacetylated SL) of samples from cultivations with C1–C10 alcohols

Besides the SL lactone as major product, some acidic open-chain SLs and the recently published hydrophilic bolaform SLs (Diao et al. 2024) could be detected by this method as early-eluting peaks at 3.4–3.8 min (Fig. 2A). LC–MS analysis confirmed the molecular ions of the bolaform congeners with varying degrees of acetylation (Table S1). Bolaform SLs were observed as a major product in cultivations without alcohol addition and in the presence of methanol or ethanol (Figs. 2A+C), but their concentration was significantly lower in the presence of middle- and long-chain alcohols (Figs. 2B+C). Long-chain C16 and C18 fatty alcohols were well tolerated by *S. bombicola,* and good overall SL production of around 60 g l^−1^ was observed in shake-flask cultures. When shorter-chain alcohols, such as butanol, hexanol or octanol, were added to the culture, little or no further SL production was observed. Surprisingly, the toxicity of methanol and ethanol appeared to be much lower and the total SL production was comparable to that of the reference cultivation without alcohol.

The addition of alcohols resulted in new signals in HPLC-ELSD analysis with the SL methyl ester eluting slightly earlier than the lactone, indicating a slightly higher hydrophilicity, whereas longer-chain alcohols produced signals at progressively longer retention times, consistent with increasing hydrophobicity (Figs. 2A+B, S5). To verify ester formation, LC–MS analysis was performed and the expected molecular ions of the diacetylated esters [M-H]^−^ (Fig. S6) as well as the corresponding formate adduct ions [M+CHO_2_]^−^ (Fig. 2D, Table S1) were detected for all alcohols by negative-mode APCI-MS. The SL methyl ester was purified by flash chromatography to elucidate its structure by NMR spectroscopy in comparison to the SL lactone (Tables 1, S2). A signal at δ 5.17–5.18, corresponding to the 4″-OH group, is absent only in the lactonic SL, and the HMBC correlation between H-4″ of the sophorose and C-1 of the fatty acid is likewise observed only in the lactone. Both findings support opening of the ring upon esterification. Additional signals corresponding to the methyl group were present only in the SL ester. The position of esterification is further supported by HMBC correlations between the protons adjacent to the hydroxy group of the alcohol and C-1 of the fatty acid.

**Table 1:** ^1^H and ^13^C NMR data of lactonic SL, SL methyl ester, SL isobutyl ester from *S. bombicola* (grey rows) as well as SL ethyl ester and SL isopropyl ester from S*. kuoi* (white rows); the complete signal list is provided in Table S2

Position	δH [ppm] (mult.)/δC [ppm]					Key correlation
	Lactone	Methyl	Ethyl	Isopropyl	Isobutyl	
*Fatty acid*						
C-16	1.48, 1.37 (m)/37.3	1.48, 1.30 (m)/36.9	1.27 (m)/26.0	1.25 (m)/26.0	1.48, 1.30 (m)/37.0	ω-1 vs ω regio-isomer visible
C-17	3.62 (m)/77.9	3.61 (m)/76.8	1.48 (m)/29.5	1.48 (m)/29.5	3.61 (m)/76.4	ω-1 vs ω regio-isomer visible
C-18	1.12 (d)/21.8	1.09 (d)/21.8	3.68, 3.45/(m) 69.2	3.68, 3.45/(m) 69.2	1.10 (d)/21.8	ω-1 vs ω regio-isomer visible
*Sophorose*						
C-1'	4.37 (d)/102.1	4.36 (d)/101.1	4.32 (d)/101.4	4.32 (d)/101.4	4.36 (d)/101.4	HMBC H-1' → C-17 (ω-1)/C-18 (ω): glycosidic bond to the fatty acid
C-4″	4.71 (t)/70.8	3.10 (m)/70.2	3.11 (m)/70.2	3.15 (m)/70.2	3.08 (m)/70.2	HMBC H-4'' → C-1 in the lactone: 1,4''-macrolactone ring
4″-OH	–	5.17	5.18	5.18	5.17	present only in esters: confirms absence of lactone
*Ester*						
O–CH_3_/O–CH_2_/O–CH	–	3.57 (s)/51.5	4.04 (q)/60.2	4.87 (hept)/67.3	3.80 (d)/69.8	HMBC → C-1: identifies the ester in each case
CH (isobutyl)	–	–	–	–	1.85 (hept)/27.9	
CH_3_ (ester)	–	–	1.17 (t) 14.7	1.17 (d) 22.1	0.88 (d) 19.6	

### SL ester synthesis with primary alcohols: optimization strategies

Under initial reaction conditions, ratios of SL ester to lactonic SL were low for all short- and middle-chain alcohols. Thus, optimization of cultivation conditions was needed to improve ester yields. As a starting point, the concentration of yeast extract in the production medium was increased from 1 to 3 g l^−1^ to increase biomass concentration. A notable effect was a significant increase in SL production rate, with SL titers reaching 20–40 g l^−1^ after 3 days compared to 7–10 days under the initial conditions.

**Fig. 3:**
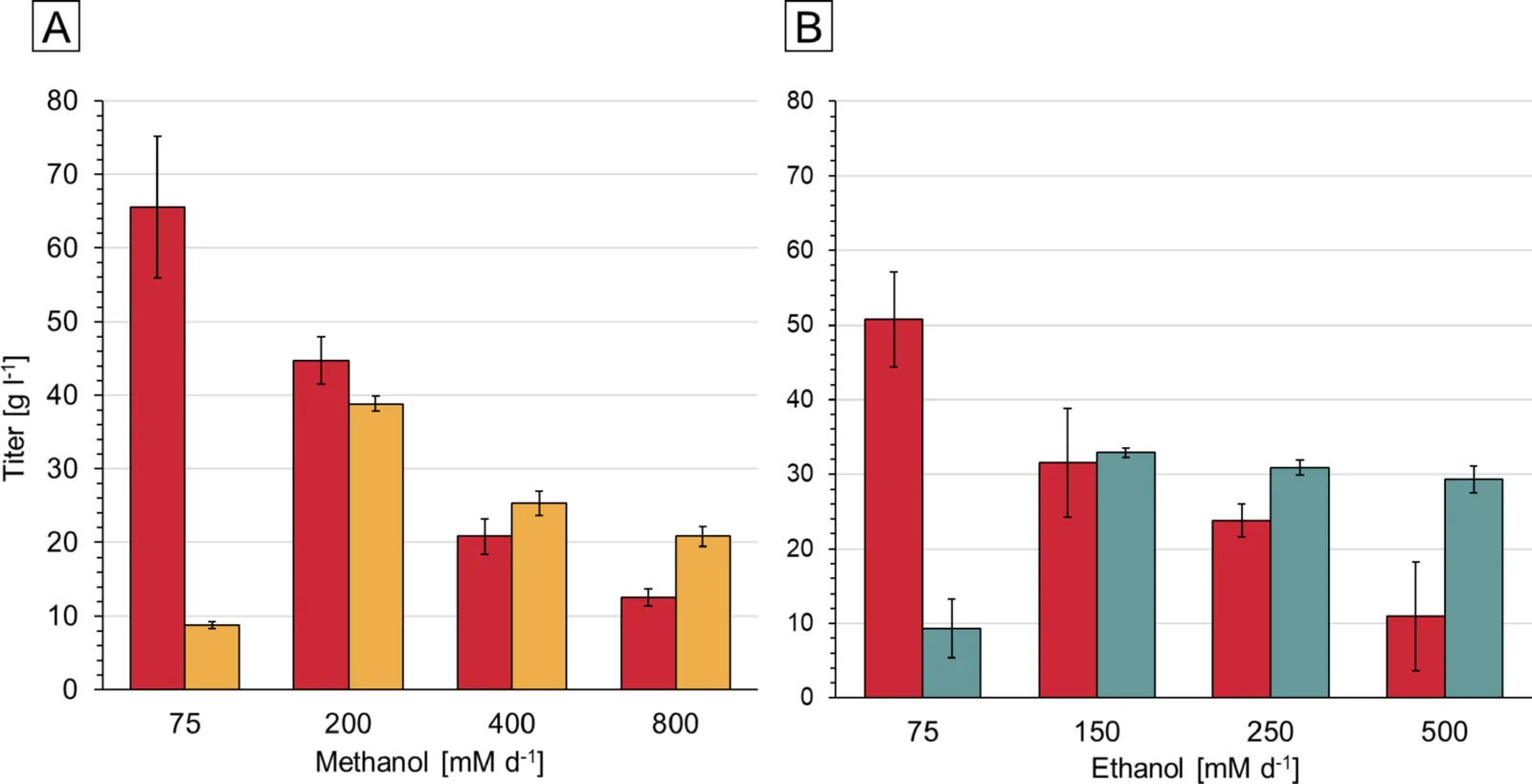
Effect of alcohol feeding rates on lactonic SL and SL ester production in *S. bombicola* cultivations supplemented with methanol (**A**) and ethanol (**B**); red, yellow and cyan bars represent the lactonic SL, SL methyl ester and SL ethyl ester respectively

To further improve ester yields, alcohol feeding was investigated as a key parameter with methanol and ethanol as the least toxic substrates. Feeding rates of 75–800 mM d^−1^ for methanol and 75–500 mM d^−1^ for ethanol were comparatively analyzed (Fig. 3, Table S3). Ester fractions of 52% were achieved at alcohol feeding rates of 800 mM d^−1^ for methanol and 47% at 500 mM d^−1^ for ethanol. However, increasing the alcohol feeding rate resulted in a decrease of total SL titers from 109 down to 40 g l^−1^ in the case of methanol and from 76 down to 62 g l^−1^ for ethanol (Table S3). At the same time a shift towards a higher SL ester to SL lactone ratio was observed (Fig. 3).

### SL ester formation with secondary and branched alcohols

**Fig. 4:**
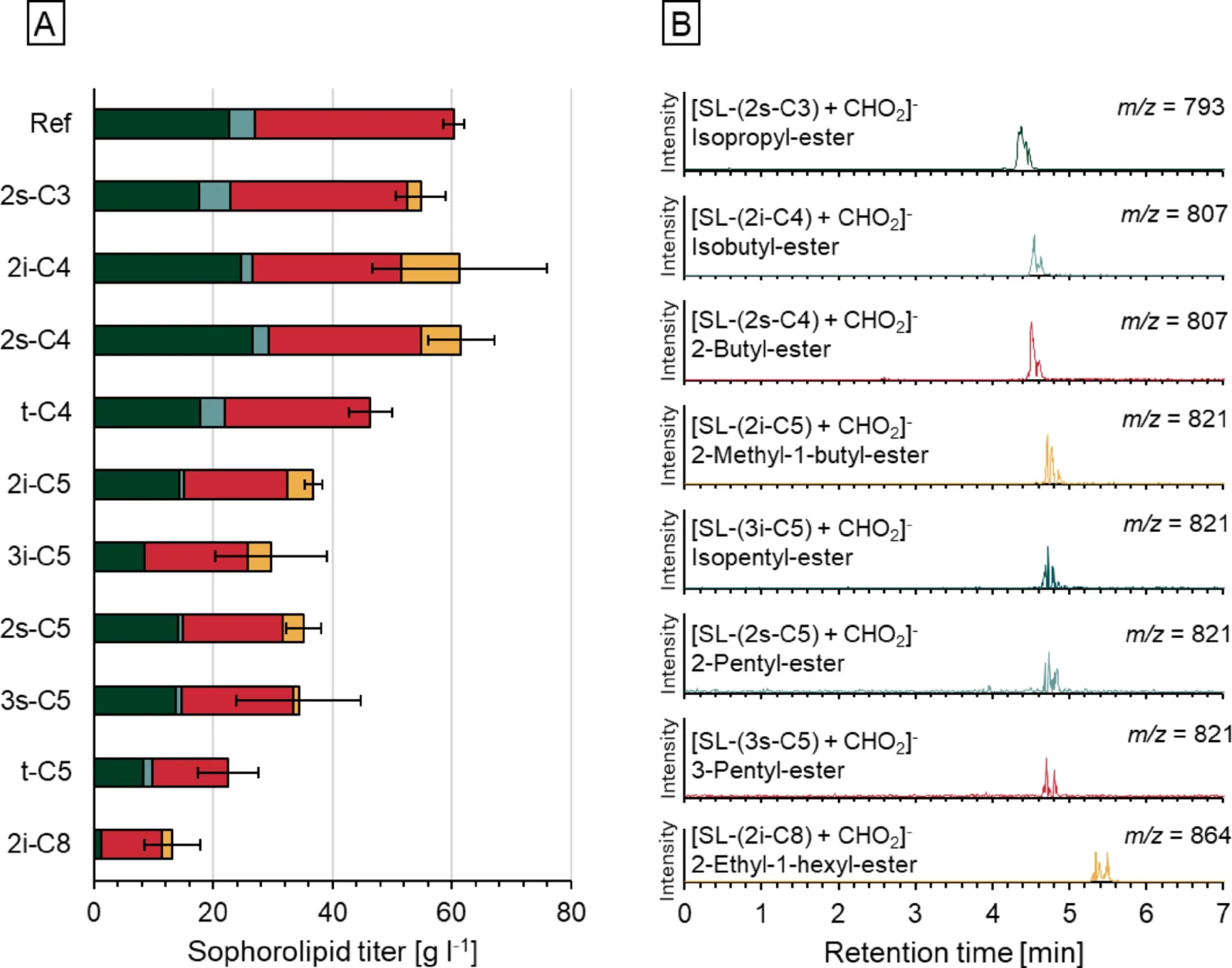
**A** SL titers and species analysis of cultivations in the presence of 1 g l^−1^ yeast extract and 150 mM of branched, secondary and tertiary alcohols in comparison to reference cultivation without alcohol supplementation; from top to bottom isopropanol (2s-C3), isobutanol (2i-C4), 2-butanol (2s-C4), *tert*-butanol (t-C4), 2-methyl-1-butanol (2i-C5), 3-methyl-1-butanol (3i-C5), 2-pentanol (2s-C5), 3-pentanol (3s-C5), *tert*-pentanol (t-C5) and 2-ethyl-1-hexanol (2i-C8); green, cyan, red and yellow bars represent bolaform, open chain, lactonic and esterified SL respectively; **B** Overlay of extracted ion chromatograms (EICs) for SL ester [M+CHO_2_]^−^ molecular ions (*m/z* values for diacetylated SL)

Having demonstrated the successful incorporation of all tested primary alcohols into SL esters, we were interested whether more complex alcohols would be within the substrate scope for transesterification. To systematically elucidate the substrate tolerance of *S. bombicola*, secondary, tertiary, and branched alcohols were examined under the conditions established for the primary alcohols. SL production followed a similar trend as with the primary alcohols, with toxicity generally increasing with chain length (Figs. 4A, S7). Interestingly, the secondary, tertiary, and branched alcohols appeared to be less toxic than their primary alcohol counterparts. Notably, isopropanol and C4 alcohols were well tolerated, resulting in SL titers of around 60 g l^−1^, comparable to those of the reference cultivation without alcohol supplementation.

The formation of esters was monitored by LC–MS searching for the respective molecular ions of the diacetylated esters [M-H]^−^ (Table S1, Fig. S8) and the corresponding formate adducts [M+CHO_2_]^−^ (Fig. 4B, Table S1). SL esters with all secondary and branched-chain alcohols could be detected, whereas the tertiary alcohols were not incorporated into SLs. The isobutyl ester was analyzed exemplarily by NMR spectroscopy after chromatographic purification. The signals of the SL lipid and sophorose moiety were identical to those of the SL methyl ester, and additional signals of the isobutyl headgroup could be assigned (Tables 1, S2), confirming the diacetylated isobutyl ester structure.

### Optimization and scale up of SL ester production with secondary and branched alcohols

**Fig. 5:**
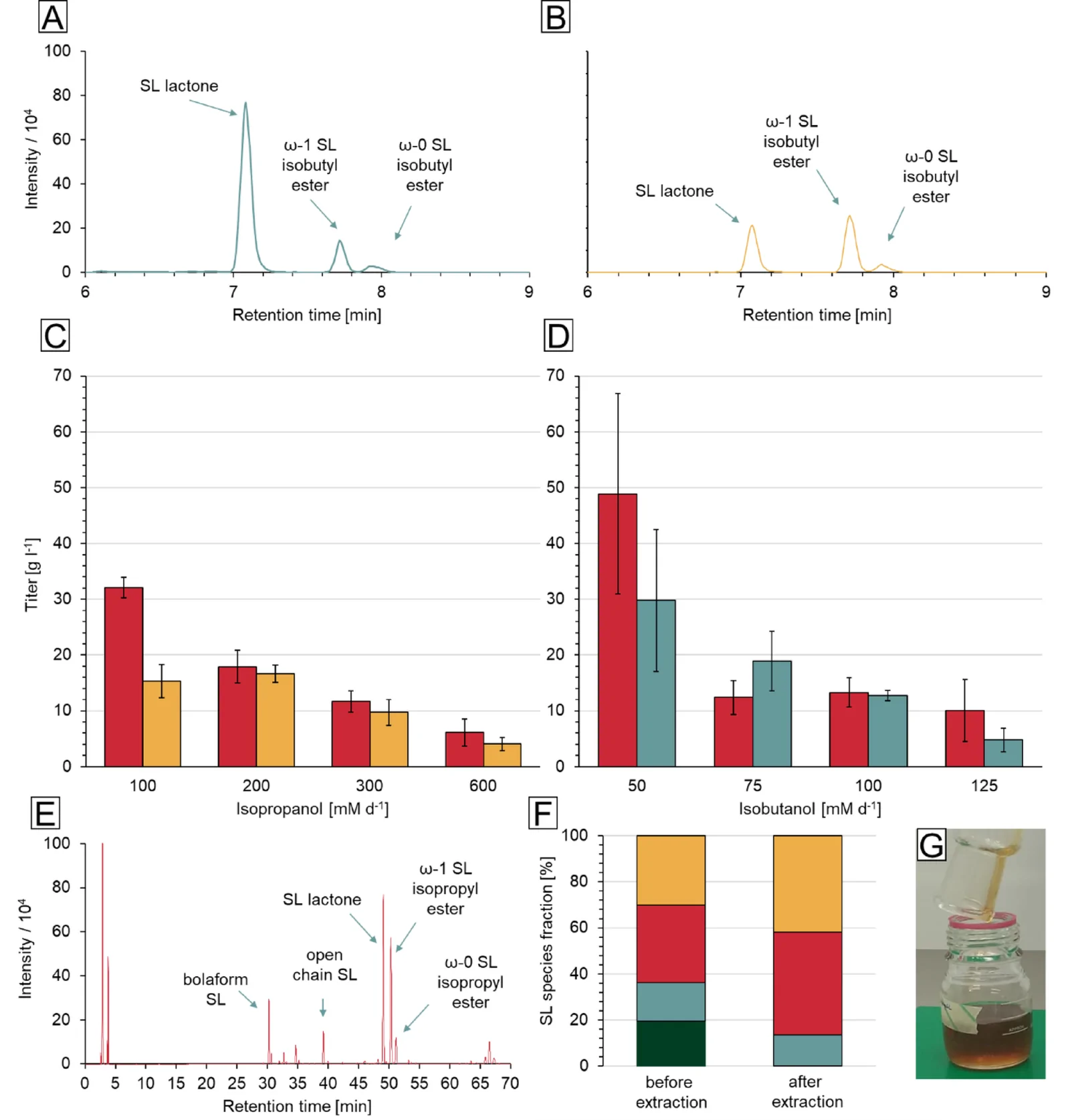
**A** + **B** HPLC-ELSD chromatograms of *S. bombicola* culture broth after a single addition of 150 mM isobutanol (**A**) and after repeated daily supplementation of 75 mM d^−1^ isobutanol (**B**); **C** + **D** Effect of the isopropanol (**C**) and isobutanol (**D**) feeding rate on the titers of SL lactone (red) and SL ester (yellow, cyan) formation; **E**–**G** Scale-up of SL isopropyl ester synthesis in a 2 l fermentation with an isopropanol feeding of 200 mM d^−1^: HPLC chromatogram of the culture broth at the end of cultivation (**E**), SL product composition in the fermentation broth and after gravity separation and extraction with green = bolaform SL, cyan = open chain SL, red = SL lactone and yellow = SL isopropyl ester (**F**), and the isolated product mixture after separation from the culture medium (**G**)

Isopropanol and isobutanol were selected for optimization of SL ester synthesis. With isobutanol, a maximum ester fraction of 45% was achieved, whereas only 36% were reached with isopropanol (Table S3). Increasing the feeding rates lowered total SL formation from approximately 68 to 12 g l^−1^ for isopropanol and 94 to 17 g l^−1^ for isobutanol (Table S3). Consistent with the optimization strategies applied for methanol and ethanol, substrate feeding was successfully employed to increase the SL ester to SL lactone ratio (Fig. 5A–D). Suitable feeding rates with a high ester fraction and sufficient SL titers were found to be in the range of 200 mM d^−1^ for isopropanol and 75 mM d^−1^ for isobutanol.

Scale-up of SL ester production in a 2.5 l bioreactor was tested with isopropanol applying the optimized feeding rate of 200 mM d^−1^ obtained from shake flask culturing (Figs. 5E–G, S9). The fermentation was run for 14 days and the final isopropyl ester fraction was comparable to the shake-flask experiment being 30% of all SL species, which corresponds to 47% when only lactonic SLs and isopropyl esters are considered. The total SL titer reached nearly 100 g l^−1^, which was a two-fold increase over the shake-flask cultures and corresponds to 60 g of SL isopropyl ester in the 2 l fermentation volume. The product recovered from the gravimetrically separated heavy SL-rich bottom phase after extraction and solvent removal was a viscous brown syrup that had a comparable appearance and consistency of the crude product of a conventional SL lactone fermentation (Fig. 5G). After flash chromatography and crystallization from acetone/hexane, the individual SL esters were obtained as odorless white crystalline solids.

### SL ester production capabilities of different species of the *Starmerella* clade

To assess whether SL ester formation is a capability conserved among SL-producing yeasts, the feeding experiments were conducted with five additional members of the *Starmerella* clade including *S.* *batistae*, *S. caucasica*, *S. davenportii*, *S. riodocensis* and *S. kuoi*. All strains were capable of producing SL species (Fig. S10) and cultivation of *S. caucasica* and *S. davenportii* in the presence of oleic acid showed that they mainly yielded open-chain SLs with varying degrees of acetylation. In both HPLC-ELSD and LC–MS, the diacetylated and non-acetylated open-chain congeners gave the most intense signals, and minor amounts of bolaform SLs were also detected. Cultivation of *S. batistae*, *S. riodocensis* and *S. kuoi* in the presence of oleic acid gave predominantly diacetylated open-chain SLs.

**Fig. 6:**
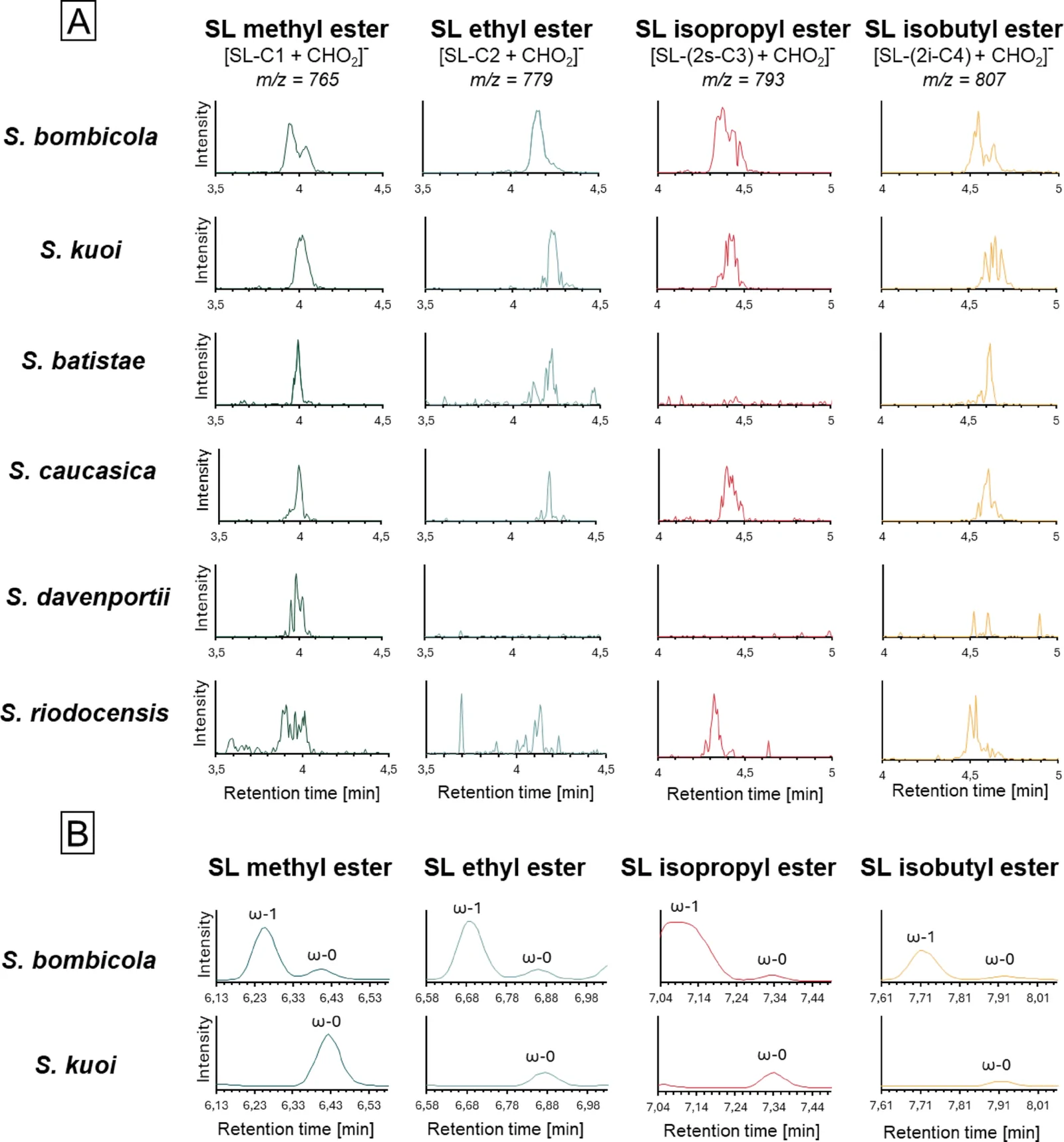
**A** Extracted ion chromatograms of SL ester [M+CHO_2_]^−^ molecular ions after feeding methanol, ethanol, isopropanol or isobutanol to the six *Starmerella* species; intensity scales were not normalized and differ between panels; **B** Comparison of SL esters produced with *S. bombicola* (upper trace) and *S. kuoi* (lower trace) with methanol (C1), ethanol (C2), isopropanol (2s-C3) and isobutanol (2i-C4) as substrates (enlarged details of the HPLC-ELSD chromatograms within the retention time of the respective SL ester are shown)

A selection of alcohols was tested with all species covering linear primary (methanol and ethanol), secondary (isopropanol) and branched primary alcohols (isobutanol). LC–MS analysis of the crude reaction mixtures gave signals at the *m/z* values expected for the formate adducts of the diacetylated SL esters (Fig. 6A). Well-defined peaks were obtained for all four alcohols with *S. bombicola*, *S. kuoi* and *S. caucasica*, and for the isopropyl and isobutyl esters with *S. riodocensis*. For the *S.* *riodocensis* methyl and ethyl esters, signals at the expected *m/z* were close to baseline, and these assignments should be regarded as tentative. With *S. batistae*, the methyl, ethyl and isobutyl esters were detected, whereas no signal above baseline was observed for the isopropyl ester (*m/z* 793) under the conditions tested. *S. davenportii* gave a clear peak only for the methyl ester. *S. caucasica* and *S. davenportii*, which formed non-acetylated SLs, also yielded some non-acetylated SL esters (Fig. S11).

*S. kuoi*, *S. batistae* and *S. caucasica* gave single, later-eluting signals, whereas *S. bombicola* and *S. riodocensis* gave predominantly an earlier-eluting one (Figs. 6A+B). The differences in elution pattern are caused by the formation of regioisomers differing in their hydroxylation pattern, which was verified by NMR spectroscopy of SL esters from *S. bombicola* and *S. kuoi* was. Clear differences in the chemical shifts of the C-16 to C-18 positions of the fatty acid tail are visible in both the ^1^H and ^13^C spectra (Tables 1, S2). In addition, a doublet at the C-18 position was observed only for the SLs produced by *S. bombicola*. HMBC experiments further supported the different positions of the glycosidic linkage, with H-1′ of the inner glucose correlating to C-17 in the *S. bombicola* products and to C-18 in those of *S. kuoi*. The additional signals of the ethyl and the isopropyl group were visible confirming the formation of the respective SL alkyl esters with either ω-1 hydroxylation in *S. bombicola* and ω-hydroxylation in *S. kuoi.*

## Discussion

The three-component feeding strategy presented here provides direct access to a broad range of acetylated SL esters in a single fermentation step. Glycerol was reported to be incorporated into SL structured and reacts at all hydroxyl positions (Kobayashi et al. 2023, 2024), indicating that secondary alcohols can also be accepted for the transesterification. This could be proven successfully with e.g. isopropanol. The NMR analysis of the short-chain SL esters is in accordance with the previously published structures obtained with long-chain alcohols (Tulloch and Spencer 1972; Zerhusen et al. 2020). Acetylated SL esters have so far been accessible only through a three-stage sequence: fermentative production of SL lactone, chemical transesterification with the desired acyl donor, and enzymatic re-acetylation of the sophorose head group (Bisht et al. 1999; Koh et al. 2017). These post-fermentation steps can be avoided, because the transesterification occurs in vivo without deacetylation.

For primary and secondary alcohols, alcohol toxicity rather than substrate tolerance was shown to be the limiting factor. Straight-chain alcohols of medium chain length (C3–C12) inhibited SL production under the conditions tested, whereas methanol, ethanol and the previously reported long-chain alcohols (Zerhusen et al. 2020) were well tolerated. Significant amounts of bolaform SL accumulated only in the cultures without alcohol addition and with methanol or ethanol as substrates. Low concentration of bolaform SLs with middle-chain alcohols may be explained by impaired de novo synthesis of bolaform SLs, while long-chain alcohols are well accepted indicating a faster bolaform SL transesterification. At equal carbon number, secondary and branched alcohols were less inhibitory than their linear counterparts. For example, n-propanol and n-butanol decreased SL production, while the branched, secondary and tertiary C4 alcohols and isopropanol matched the reference titers of about 60 g l^−1^. Within this group toxicity still rose with chain length, with 2-ethyl-1-hexanol showing the highest inhibition.

The inhibitory effects of the alcohols are related to their hydrophobicity. The bilayer-modifying potency of n-alcohols scales with their octanol/water partition coefficient up to a cut-off chain length (Ingólfsson and Andersen 2011), and branching or an internal hydroxy group lowers that coefficient relative to the linear isomer. The trend of branched alcohols being less inhibitory than their straight-chain counterpart of equal carbon number is consistent with findings in *E. coli* (Wilbanks and Trinh 2017). The lower toxicity of longer-chain alcohols is explained by their limited solubility, which prevents them from reaching inhibitory concentrations. The high toxicity of medium chain alcohols to yeasts is mainly caused by impairing membrane function and cause a common problem, when alcohols were applied for fermentation broth extraction (Offeman et al. 2008) or in the design of medium-chain alcohol producing strains for e.g. biofuel applications (Davis López et al. 2018; Azambuja and Goldbeck 2020).

Increasing the concentration of short-chain alcohols in feeding trials led to a higher SL ester share but reduction of overall SL titers. Daily methanol supplementation of 400 mM, corresponding to about 1.5% (v/v), reduced SL titer by 47%, close to the 56% reduction observed by Ashby and Solaiman at comparable methanol concentrations (Ashby and Solaiman 2010). In accordance to their study, the ratio of open-chain to lactonic SLs increased, reaching 22% at 1.5% and 33% at 3% methanol. However, in our experiments, this shift was caused by a decreased formation of lactonic SL and a shift towards SL methyl ester synthesis. SL methyl esters were not found by Ashby and Solaiman and a reason for this may be the differences in the different alcohol feeding regime. The optimal feeding rates differed by roughly an order of magnitude for methanol, ethanol, isopropanol and isobutanol. Volatility alone is unlikely to explain this trend and differences in metabolic fate, intrinsic toxicity, substrate preference of SBLE and partitioning of the more hydrophobic alcohols into the SL-rich phase may all contribute. A similar partitioning effect could be observed in lipase catalyzed esterification in a two-phase system with hydrophilic deep eutectic solvents and a separate lipid phase (Kleiner and Schörken 2015). Transfer of the isopropyl ester process to the 2 l scale preserved the ester fraction at equal feeding rate while approximately doubling the total titer, indicating that the process is scalable. The crude SL ester mixture formed a separate heavy phase, which is also observed in SL lactone fermentations. Thus, it can be concluded that established downstream procedures could be utilized for an industrial-scale SL ester production.

The alcohol substrate spectrum provides indirect evidence for the enzyme responsible for SL ester formation. All primary, secondary and branched alcohols tested were incorporated, while no tertiary alcohols were accepted as substrates. This characteristic is common for most lipases and esterases, and only a few of them can accept tertiary alcohols (Henke et al. 2002; Wagner et al. 2024). The relatively lower ester fraction with isopropanol fits to the same pattern, given the general preference of lipases for primary alcohols (Kuo and Parkin 1993; Souza et al. 2018). The lactone transesterase SBLE shares some identity with lipases and possesses an α/β-hydrolase fold and the GYSGGA active site motif characteristic for lipases (Ciesielska et al. 2014). The enzyme was shown to catalyze the transesterification of bolaform SLs to the lactone and accepts acylglycerides as well (Diao et al. 2024; Roelants et al. 2024; Bovijn et al. 2024). Thus, it is most probable that the same enzyme is responsible for the transesterification with alcohols, although a minor contribution of SL lactone alcoholysis cannot be excluded in our experiments. A direct proof of SBLE catalysis is still needed, and SBLE deficient or overexpressing *S. bombicola* mutants (Roelants et al. 2016) may be employed for this task.

Feeding of the corresponding fatty acid esters, in contrast, has largely failed to yield SL esters. Methyl esters such as biodiesel or methyl erucate produced only minor amounts of SL methyl ester alongside a product spectrum dominated by acidic SLs, while feeding of ethyl and propyl esters of soybean oil did not produce detectable levels of the corresponding SL esters (Ashby et al. 2005, 2006; Shin et al. 2010; Kim et al. 2020). Most probably, the alcohol is released intracellularly during the glycosylation steps to produce the bolaform SL, and is not present in large enough amounts as substrate for the transesterification step with SBLE, which takes place extracellularly (Ciesielska et al. 2014). Supplying the alcohol as a separate feed component decouples it from the lipid substrate and makes it an independent process variable, which allowed us to synthesize a wide SL ester product spectrum.

The feeding experiments were expanded to five members of the *Starmerella* clade including *S. caucasica and S. davenportii*, which have not been tested for SL production before. SL production by these two species was considered likely, given the close relationship of *S. caucasica* to *S. bombicola* and *S. kuoi* (Sipiczki 2013; Vandeputte et al. 2025) and the phylogenetic relationship of *S. davenportii* to the known SL-producing species *S. stellata* (Kurtzman et al. 2010; Timouma et al. 2026). In accordance, SL production was verified with both species. In contrast to the lactone-forming *S.* *bombicola*, the yeasts *S. batistae, S. riodocensis* and *S. kuoi* have been reported to produce predominantly open-chain acidic SLs (Konishi et al. 2008; Price et al. 2012; Intasit and Soontorngun 2023) and were chosen to elucidate whether (trans)esterification with alcohols is a general property of the *Starmerella* clade. *S. kuoi* is of particular interest, as it forms exclusively open-chain SLs linked via an ω-hydroxylated fatty acid (Kurtzman 2012; Bollmann et al. 2019).

All species tested were capable of synthesizing SL esters and the retention-time shifts observed between species corresponded to the reported ω and ω-1 hydroxylation preferences (Konishi et al. 2008; Price et al. 2012). Thus, all species enable the direct access to SL esters, a compound class with promising surface-active and bioactive properties (Koh et al. 2017; Totsingan et al. 2021; Miceli et al. 2022; Roberge et al. 2023). SL lactone formation seems not to be a prerequisite for SL ester formation as was reported for *S. kuoi* before (Zerhusen et al. 2020). An SBLE homolog was identified in the genome of *S. kuoi* (Bovijn et al. 2024), which is potentially responsible for the formation of SL dimers and oligomers (Price et al. 2012; Bollmann et al. 2019) via intermolecular transesterification.

A sequence alignment of potential SBLE homologs identified sequences with 40–50% identity and > 60% similarity to the *S. bombicola* SBLE in *S. kuoi*, *S. riodocensis* and *S. batistae* (Fig. S12, Table S4). A genome sequence of *S. caucasica* is not yet available, nevertheless, the close homology to *S. bombicola* and *S. kuoi*. suggests the existence of a similar gene sequence. In contrast, no close relative of SBLE could be identified in the genome of *S. davenportii*, and the most similar sequence showed significantly less sequence identity with SBLE than Lipase A from *Pseudozyma aphidis*, which has been described as a structurally-similar distant relative of SBLE (Ciesielska et al. 2016). An analysis of the genome of *S. stellata*, the closest known SL-producing relative of *S. davenportii*, revealed that, once again, no SBLE homolog could be identified and that the most closely related gene sequence shows a high degree of similarity to the gene segment identified in *S. davenportii*. As discussed by Timouma et al., the significant differences in the gene architectures suggests that alternative pathways for the production of sophorolipids have evolved in different *Starmerella* lineages (Timouma et al. 2026) and this seems to be the case for *S. stellata* and *S. davenportii*. In *S. davenportii* only SL methyl esters could be identified, whereas the other strains produced esters with all alcohols and this finding points to a different enzyme than an SBLE homolog being responsible for this activity.

## Conclusions

In our study, we demonstrated that tailoring of structurally diverse SL esters with varying hydrophobicity is possible in biotransformation with *S. bombicola*. Primary, secondary and branched alcohols were accepted as substrates and an alcohol dosage strategy reduced toxicity and markedly increased SL ester yields in shake-flask trials. A first scale-up was successful; further improvements may nevertheless be possible through a tighter feeding regime under continuous alcohol monitoring in the fermentation broth. Screening of five members of the *Starmerella* clade identified two new SL-producing species and revealed that the capacity for SL ester synthesis appears to be widespread among SL-producing species. Elucidating the underlying sophorolipid biosynthetic pathways and optimizing SL ester production using alternative strains represent an interesting challenge for future research.

## Supplementary Information

Below is the link to the electronic supplementary material.

Supplementary Material 1

## Data Availability

The data generated for this study are provided in the manuscript and the supplementary data file and are maintained in the Faculty of Applied Natural Sciences at TH Köln—Campus Leverkusen, Campusplatz 1, 51379 Leverkusen, Germany. In case of additional requirements, please contact the corresponding author.
