# Supplementary material for: Microbial synthesis of short-chain sophorolipid esters using yeasts of the Starmerella clade
Source: AMB Express. 2026 Oct 24;16:103. doi: 10.1186/s13568-026-02130-5 (PMC13696635; doi:10.1186/s13568-026-02130-5)
Supplement: Supplementary file 1 [file 13568_2026_2130_MOESM1_ESM.pdf]

Supplementary Data for

Microbial synthesis of short-chain sophorolipid esters using yeasts of the *Starmerella* clade

Eugen Banmann[1], Enes Medet Aksoy[1], Ulrich Schörken*,[1]

[1] TH Köln – Campus Leverkusen, Campusplatz 1, 51379 Leverkusen, Germany

***Correspondence:** Ulrich Schörken, **Email:** ulrich.schoerken@th-koeln.de

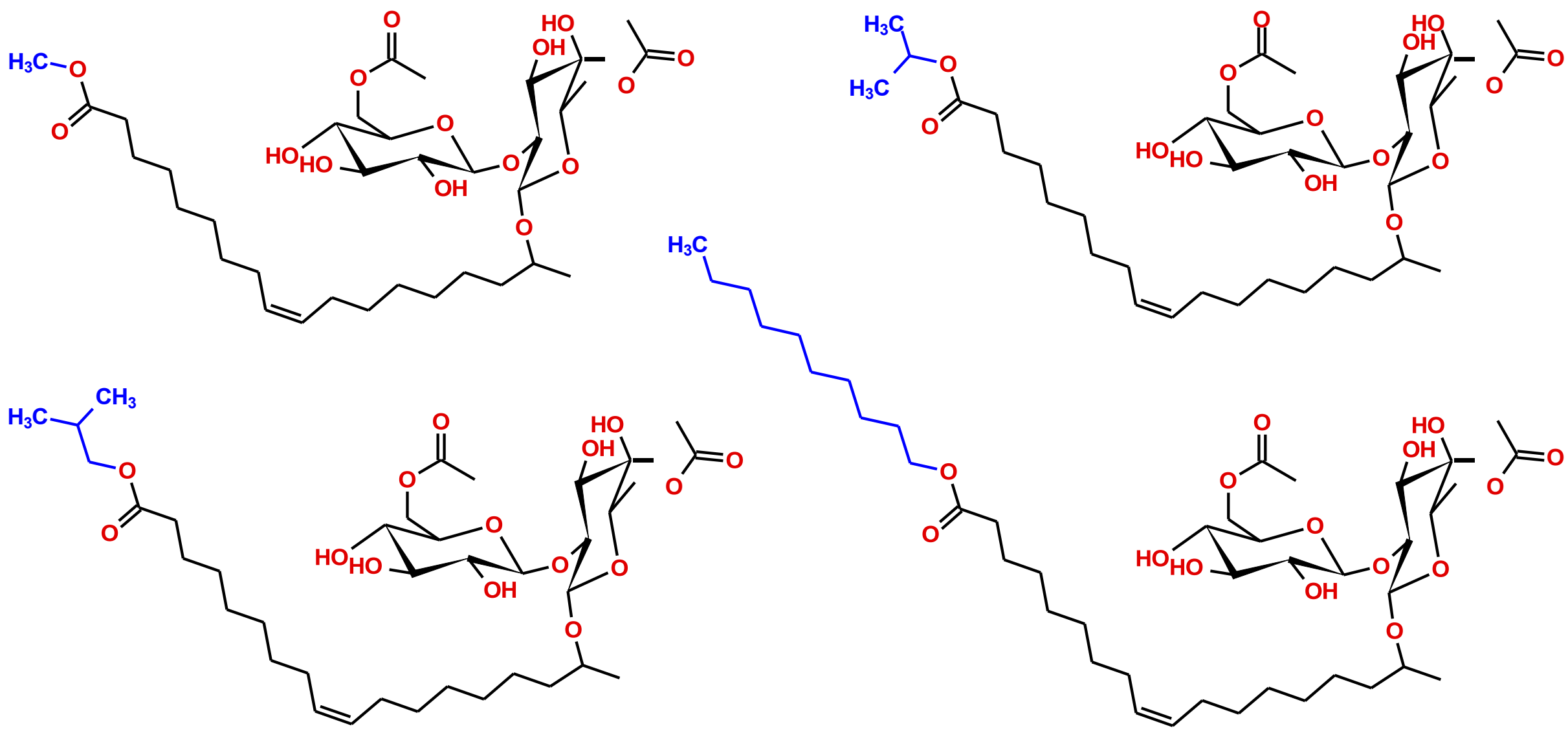

Fig. S1 Exemplary structures of diacetylated SL esters with 17-hydroxyoleic acid and methyl (upper left), isopropyl (upper right), isobutyl (lower left) and decyl (lower right) as ester groups highlighted in blue colour.

Primary straight-chain alcohols

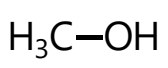

C1 Methanol

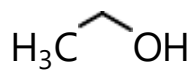

C2 Ethanol

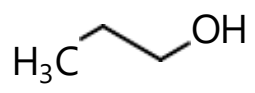

C3 Propanol (propan-1-ol)

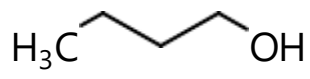

C4 Butanol (butan-1-ol)

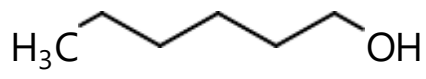

C6 Hexanol (hexan-1-ol)

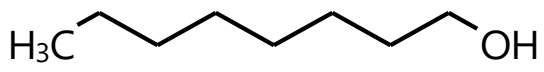

C8 Octanol (octan-1-ol)

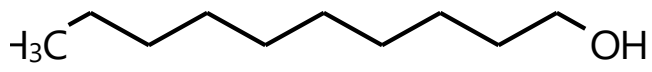

C10 Decanol (decan-1-ol)

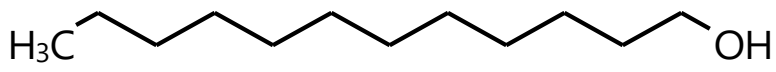

C12 Dodecanol (dodecan-1-ol)

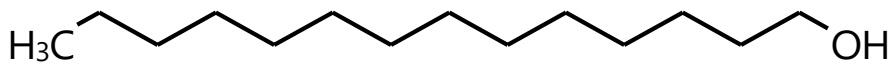

C14 Tetradecanol (tetradecan-1-ol)

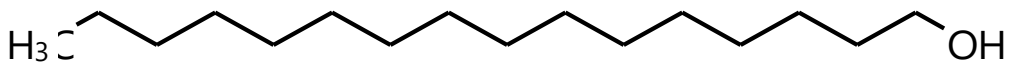

C16 Hexadecanol (hexadecan-1-ol)

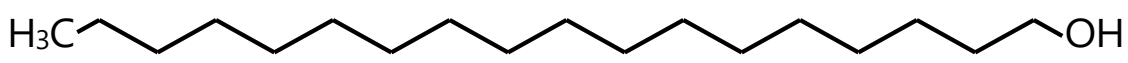

C18 Octadecanol (octadecan-1-ol)

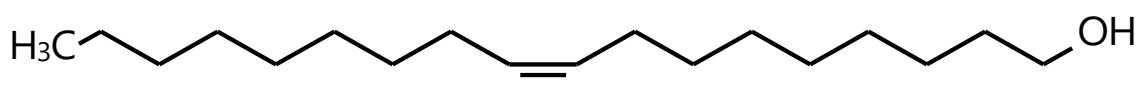

C18:1 Oleyl alcohol ((9*Z*)-octadec-9-en-1-ol)

Branched-chain primary alcohols

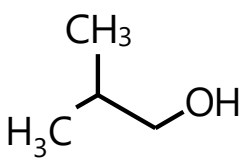

2i-C4 *iso*-Butanol
(2-methylpropan-1-ol)

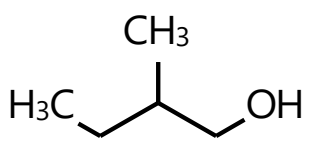

2i-C5 2-Methyl-1-butanol
(2-methylbutan-1-ol)

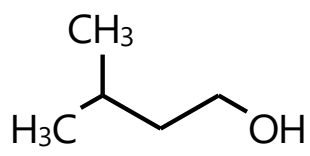

3i-C5 *iso*-Pentanol
(3-methylbutan-1-ol)

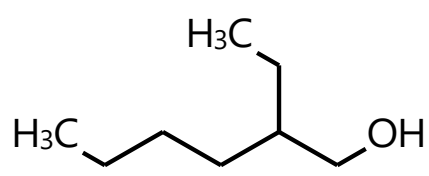

2i-C8 2-Ethyl-1-hexanol
(2-ethylhexan-1-ol)

Secondary alcohols

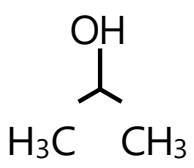

2s-C3 Isopropanol
(propan-2-ol)

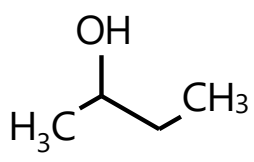

2s-C4 2-Butanol
(butan-2-ol)

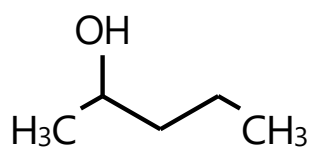

2s-C5 2-Pentanol
(pentan-2-ol)

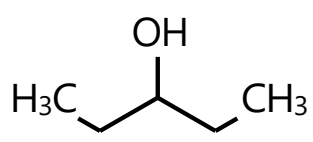

3s-C5 3-Pentanol
(pentan-3-ol)

Tertiary alcohols

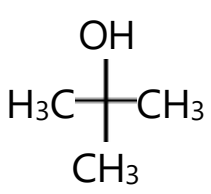

t-C4 *tert*-Butanol (2-methylpropan-2-ol)

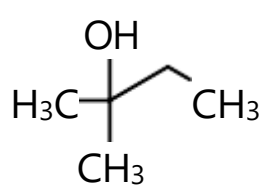

t-C5 *tert*-Pentanol (2-methylbutan-2-ol)

Fig. S2 Chemical structures of all alcohols tested as substrates for ester production with IUPAC name (grey) and abbreviation (red)

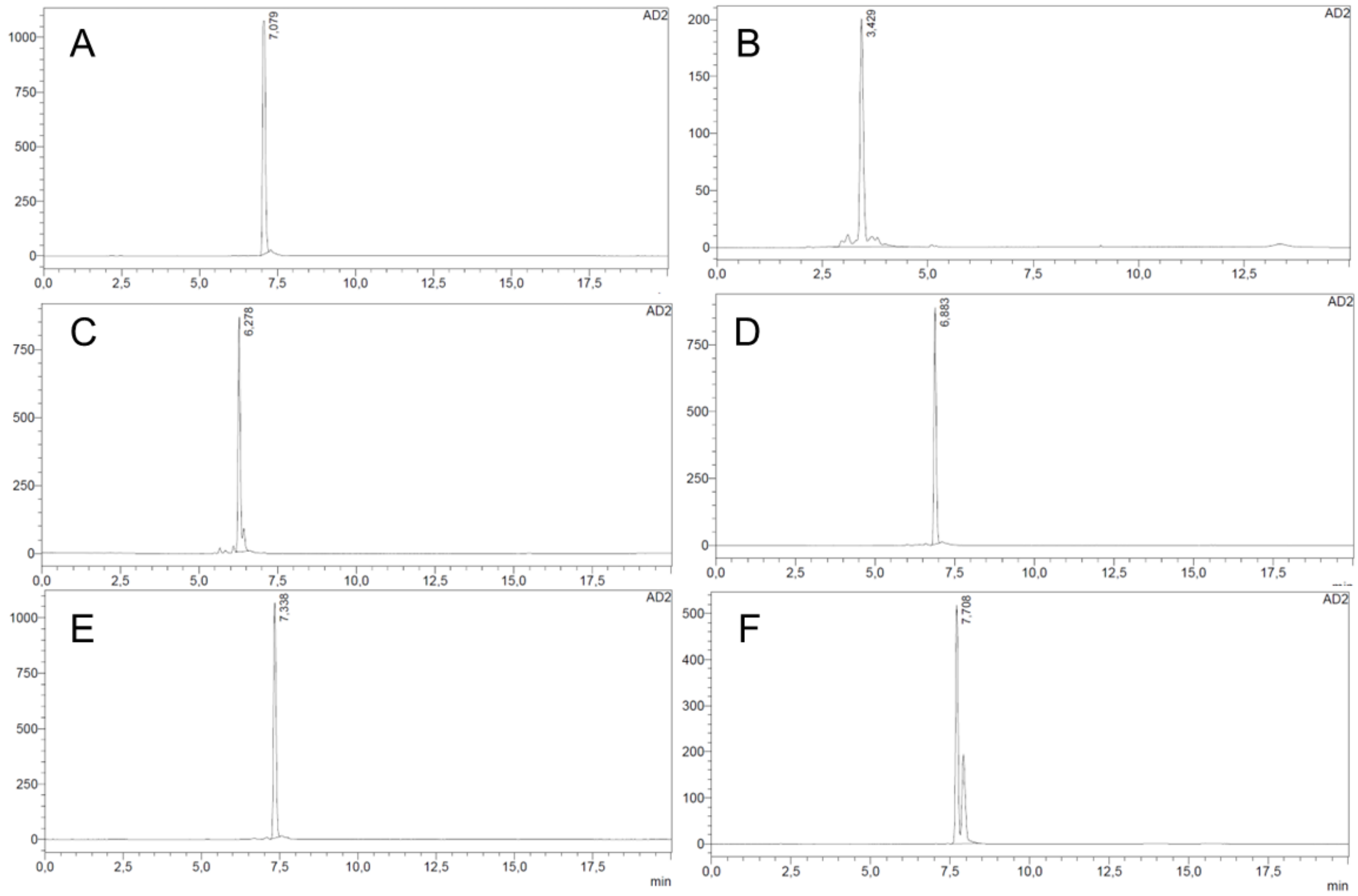

Fig. S3 LC-ELSD chromatogram of purified sophorolipid species with (A) SL lactone, (B) bolaform SL with different degrees of acetylation, (C) SL methyl ester, (D) SL ethyl ester, (E) SL isopropyl ester and (F) SL isobutyl ester (ω and ω-1)

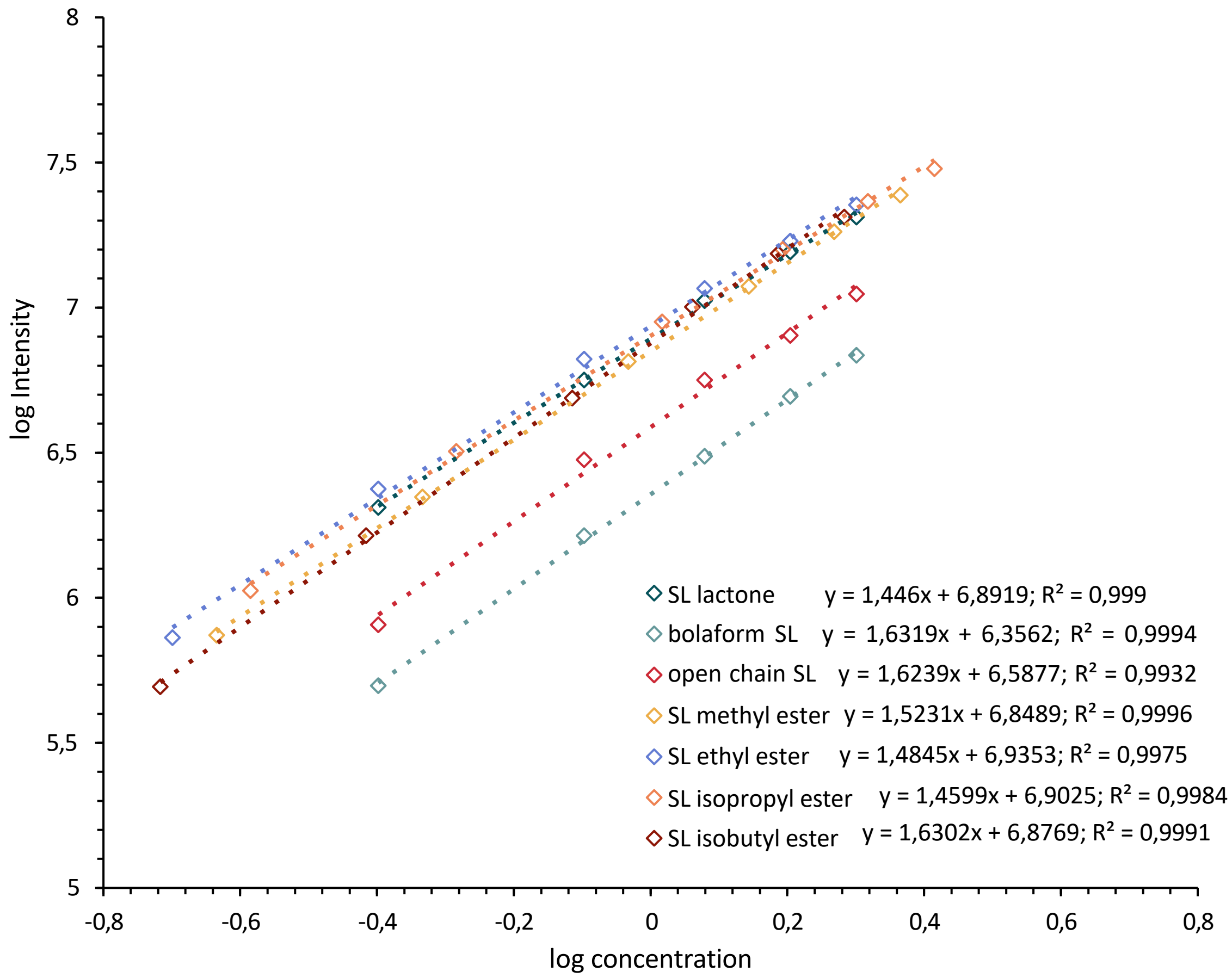

Fig. S4 Calibration curves for the quantification of different SL species by HPLC-ELSD. Peak areas and concentrations are plotted double-logarithmically. Corresponding regression equations and coefficients of determination are given in the legend.

Table S1 Retention times of SL species detected in LC-MS analysis in negative ion mode using APCI-MS including m/z values for molecule $[M-H]^-$ and formate adduct $[M+CHO_2]^-$ ions and retention times in LC-ELSD analysis with individual peak assignment for ω-1- and ω-hydroxylated species. For all ester products the major species detected is the diacetylated oleic acid-based SL with either ω- or ω-1-hydroxylation.

Species	LC-MS	m/z	m/z	LC-ELSD	LC-ELSD	Comment
	(min)	$[M-H]^-$	$[M+CHO_2]^-$	(min)	(min)	
				ω-1	ω	
**Bolafrom SL species**						
bolaform SL, 0 Ac	2.2	945	991	-	-	
bolaform SL, 1 Ac	2.4	987	1033	3.13	3.26	
bolaform SL, 2 Ac	2.6	1029	1075	3.42	3.68	major bolaform SL
bolaform SL, 3 Ac	2.8	1071	1117	3.80	3.97	
bolaform SL, 4 Ac	3.0	1113	1159	4.10	4.15	
**Acidic and lactonic SL species**						
Acidic SL	3.40	705	751	5.06	5.18	
SL lactone C16	4.13	661	707	6.77	6.81	Only visible with hexadecanol
SL lactone C18	4.25	689	735	8.05	8.05	High conc. with C18:0
SL lactone C18:1	4.25	687	733	7.06	7.06	
**SL esters with straight chain alcohols**						
SL C1-ester	4.0	719	765	6.28	6.43	
SL C2-ester	4.2	733	779	6.70	6.86	$[M-H]^-$ identical to 18:1 lactone $[M+CHO_2]^-$
SL C3-ester	4.3	747	793	7.50	7.60	
SL C4-ester	4.6	761	807	7.76	7.98	
SL C6-ester	5.1	789	835	9.01	9.23	
SL C8-ester	5.5	818	864	10.89	11.28	
SL C10-ester	6.3	846	892	13.97	13.97	No resolution of ω and ω-1
SL C12-ester	-	874	920	15.57	16.04	Extended method
SL C14-ester	-	902	948	18.66	19.13	Extended method
SL C16-ester	-	930	976	22.01	22.52	Extended method
SL C18-ester	-	958	1004	21.97	22.44	Extended method
SL C18:1 ester	-	956	1002	26.38	27.07	Extended method
**SL esters with secondary alcohols**						
SL s-C3 ester	4.4	747	793	7.08	7.33	
SL 2s-C4 ester	4.6	761	807	7.67	7.88	
SL 2s-C5 ester	4.7	775	821	8.25	8.48	
SL 3s-C5 ester	4.7	775	821	8.22	8.45	
**SL esters with tertiary alcohols**						
SL t-C4 ester	-	761	807	No product detected		
SL t-C5 ester	-	775	821	No product detected		
**SL esters with branched primary alcohols**						
SL 2i-C4 ester	4.6	761	807	7.70	7.91	
SL 2i-C5 ester	4.8	775	821	8.30	8.52	
SL 3i-C5 ester	4.8	775	821	8.23	8.45	
SL 2i-C8 ester	5.4	818	864	10.60	10.98	

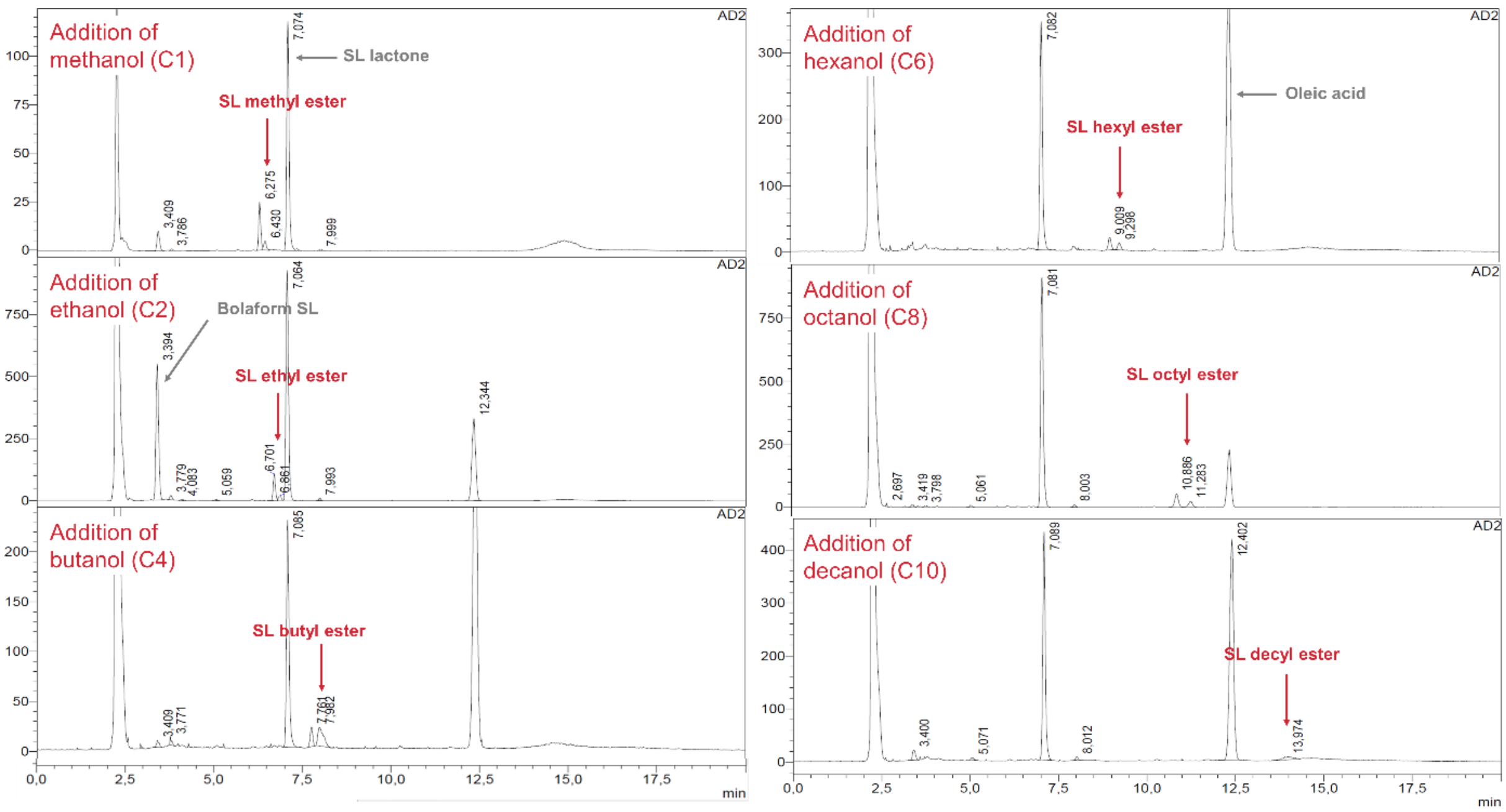

Fig. S5 HPLC-ELSD chromatograms of *S. bombicola* culture broths at the end of cultivation from alcohol screening experiments conducted under optimized conditions with 3 g l^{-1} yeast extract in the production medium.

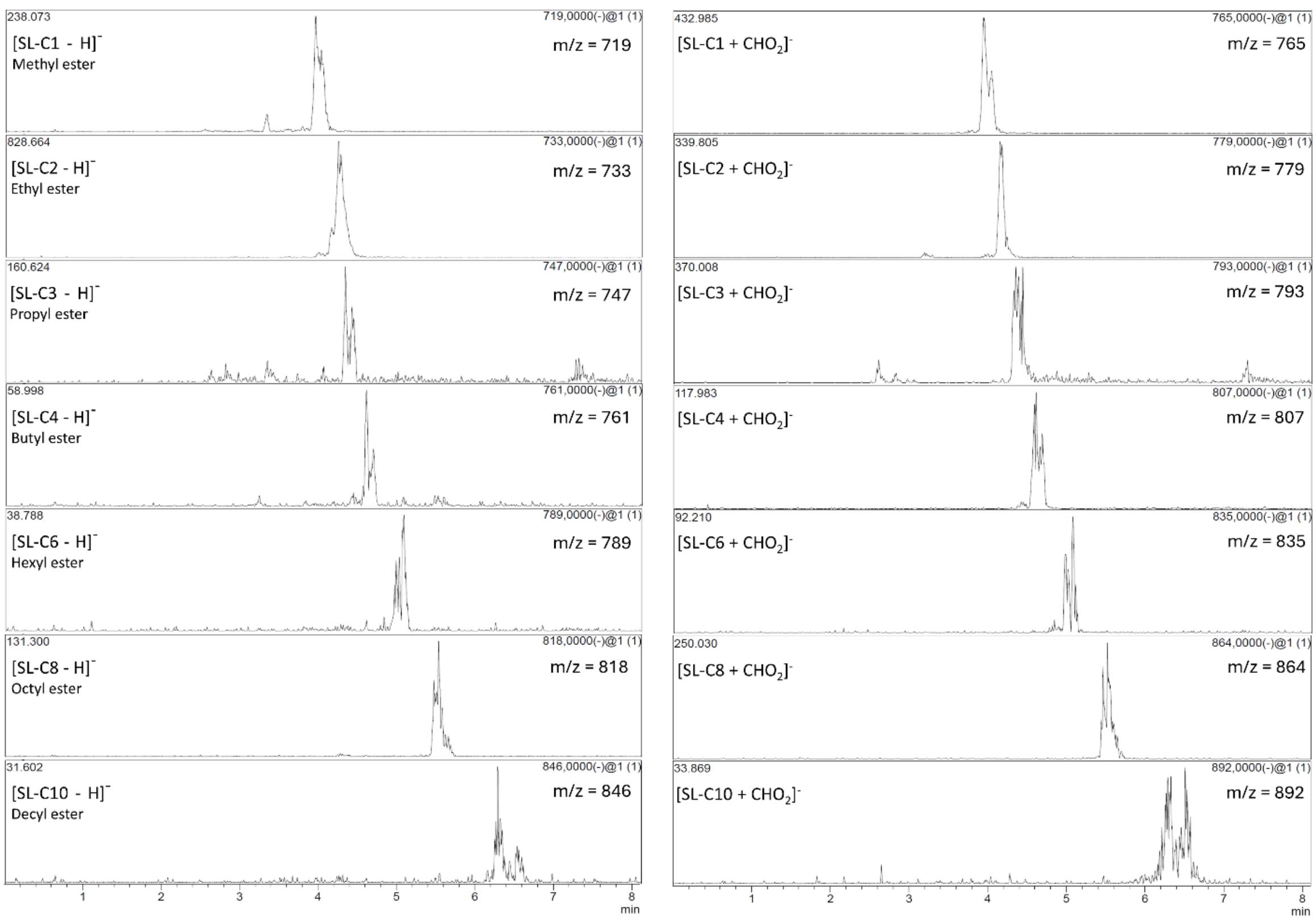

Fig. S6 Overlay of ion chromatograms (EICs) for SL ester [M – H]$^-$ ions (left) and [M + CHO_2]$^-$ ions (right) of samples from cultivations with C1 – C10 alcohols (m/z values for diacetylated SLs).

Table S2 ^{1}H and ^{13}C NMR data of lactonic SL (Lactone), SL methyl ester (Methyl), SL isobutyl ester (Isobutyl) from *S. bombicola* (designated grey) and SL ethyl ester (Ethyl) and SL isopropyl ester (Isopropyl) from S*. kuoi* (designated white).

Position	δH [ppm] (mult.)					δC [ppm]				
	Lactone	Methyl	Ethyl	Isopropyl	Isobutyl	Lactone	Methyl	Ethyl	Isopropyl	Isobutyl
**Fatty acid**										
C-1	–	–	–	–	–	172.5	173.8	173.3	172.9	173.4
C-2	2.30 (t)	2.28 (t)	2.26 (t)	2.20 (t)	2.28 (t)	34.1	33.7	34.0	34.4	34.1
C-3	1.50–1.20 (m)	1.50 (m)	1.49 (m)	1.49 (m)	1.52 (m)	24.5	25.0	24.9	25.0	25.0
C-4–C-7	1.28, 1.24 (m)	1.24 (m)	1.25 (m)	1.24 (m)	1.25 (m)	29.9–27.9	29.6–28.9	29.7–28.9	29.7–28.8	29.6–28.9
C-8	1.98 (m)	1.97 (m)	1.98 (m)	1.98 (m)	1.97 (m)	27.0	27.3	27.3	27.3	27.3
C-9	5.31 (m)	5.32 (m)	5.32 (m)	5.33 (m)	5.32 (m)	130.1	130.1	129.8	129.8	129.8
C-10	5.31 (m)	5.32 (m)	5.32 (m)	5.33 (m)	5.32 (m)	129.4	130.1	130.1	130.1	130.1
C-11	1.98 (m)	1.97 (m)	1.98 (m)	1.98 (m)	1.97 (m)	27.0	27.3	27.3	27.3	27.3
C-12–C-15	1.28, 1.24 (m)	1.24 (m)	1.25 (m)	1.24 (m)	1.25 (m)	29.9–27.9	29.6–28.9	29.7–28.9	29.7–28.8	29.6–28.9
C-16	1.48, 1.37 (m)	1.48, 1.30 (m)	1.27 (m)	1.25 (m)	1.48, 1.30 (m)	37.3	36.9	26.0	26.0	37.0
C-17	3.62 (m)	3.61 (m)	1.48 (m)	1.48 (m)	3.61 (m)	77.9	76.8	29.5	29.5	76.4
C-18	1.12 (d)	1.09 (d)	3.68, 3.45 (m)	3.68, 3.45 (m)	1.10 (d)	21.8	21.8	69.2	69.2	21.8
**Inner glucose (Glc I)**										
C-1'	4.37 (d)	4.36 (d)	4.32 (d)	4.32 (d)	4.36 (d)	102.1	101.1	101.4	101.4	101.4
C-2'	3.22 (dd)	3.20 (m)	3.21 (m)	3.23 (m)	3.20 (m)	82.7	83.4	83.1	83.1	83.4
C-3'	3.40 (m)	3.38 (m)	3.39 (m)	3.39 (m)	3.39 (m)	76.3	76.6	76.3	76.3	76.6
C-4'	3.09 (m)	3.10 (m)	3.11 (m)	3.15 (m)	3.10 (m)	70.5	70.2	70.2	70.2	70.2
C-5'	3.38 (m)	3.32 (m)	3.32 (m)	3.32 (m)	3.32 (m)	73.4	74.4	74.4	74.4	74.4
C-6'	4.24, 4.05 (dd)	4.22, 4.02 (dd)	4.23, 4.02 (dd)	4.23, 4.02 (dd)	4.22, 4.02 (dd)	64.0	64.3	64.2	64.2	64.3
**Terminal glucose (Glc II)**										
C-1''	4.51 (d)	4.40 (d)	4.40 (d)	4.40 (d)	4.40 (d)	104.3	105.0	104.7	104.7	105.0
C-2''	3.11 (m)	3.01 (m)	3.02 (m)	3.01 (m)	3.01 (m)	75.6	75.3	75.3	75.3	75.3
C-3''	3.45 (m)	3.17 (m)	3.18 (m)	3.18 (m)	3.17 (m)	73.1	76.3	76.3	76.3	76.3
C-4''	4.71 (t)	3.10 (m)	3.11 (m)	3.15 (m)	3.08 (m)	70.8	70.2	70.2	70.2	70.2
C-5''	3.59 (m)	3.37 (m)	3.37 (m)	3.37 (m)	3.37 (m)	71.8	73.7	73.7	73.7	73.7
C-6''	3.96 (m)	4.22, 4.02 (dd)	4.23, 4.02 (dd)	4.23, 4.02 (dd)	4.22, 4.02 (dd)	62.8	64.0	64.0	64.0	64.0
**Acetyl groups**										
2 × OC(O)CH₃	–	–	–	–	–	170.7, 170.3	170.9	170.7	170.7	170.7
2 × OC(O)CH₃	2.00 (s)	2.01 (s)	2.01 (s)	2.00 (s)	2.00 (s)	21.2	21.2	21.2	21.2	21.2
**Ester alkyl group**										
O-CH₃ / O-CH₂ / O-CH	–	3.57 (s)	4.04 (q)	4.87 (hept)	3.80 (d	–	51.5	60.2	67.3	69.8
CH (isobutyl)	–	–	–	–	1.85 (hept)	–	–	–	–	27.9
CH₃ (ester)	–	–	1.17 (t)	1.17 (d)	0.88 (d)	–	–	14.7	22.1	19.6
**Hydroxyl groups**										
3'-OH	5.68 (d)	5,62	5,53	5,51	5,61	–	–	–	–	–
4'-OH	5,32	5,3	5,33	5,32	5,33	–	–	–	–	–
2''-OH	5.43 (d)	5,45	5,33	5,35	5,46	–	–	–	–	–
3''-OH	5,3	5.10	5.10	5.09	5.10	–	–	–	–	–
4''-OH	-	5,17	5,18	5,18	5,17	–	–	–	–	–

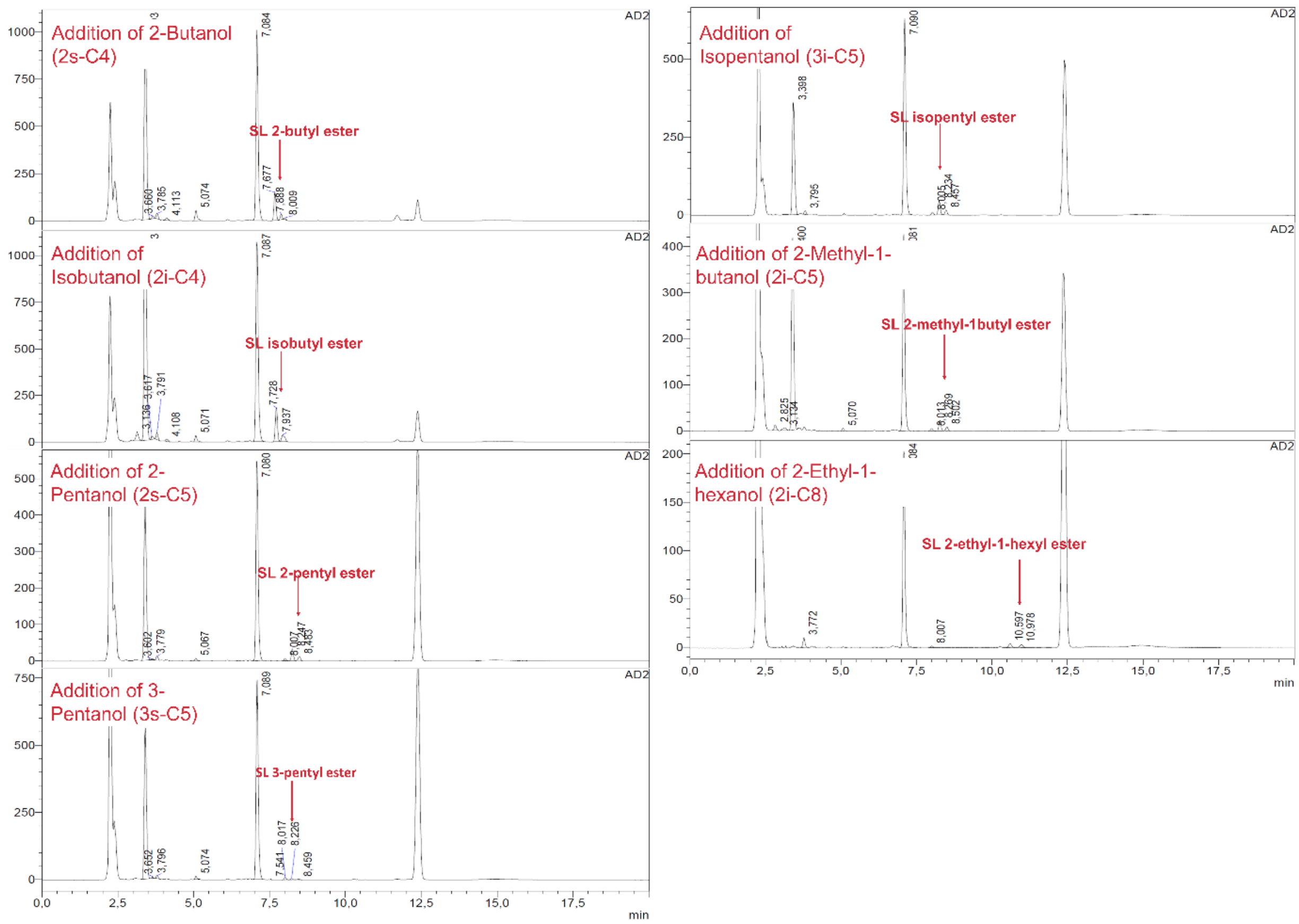

Fig. S7 HPLC-ELSD chromatograms of *S. bombicola* culture broths at the end of cultivation from secondary and branched alcohol screening experiments conducted with 3 g l^{-1} yeast extract in the production medium.

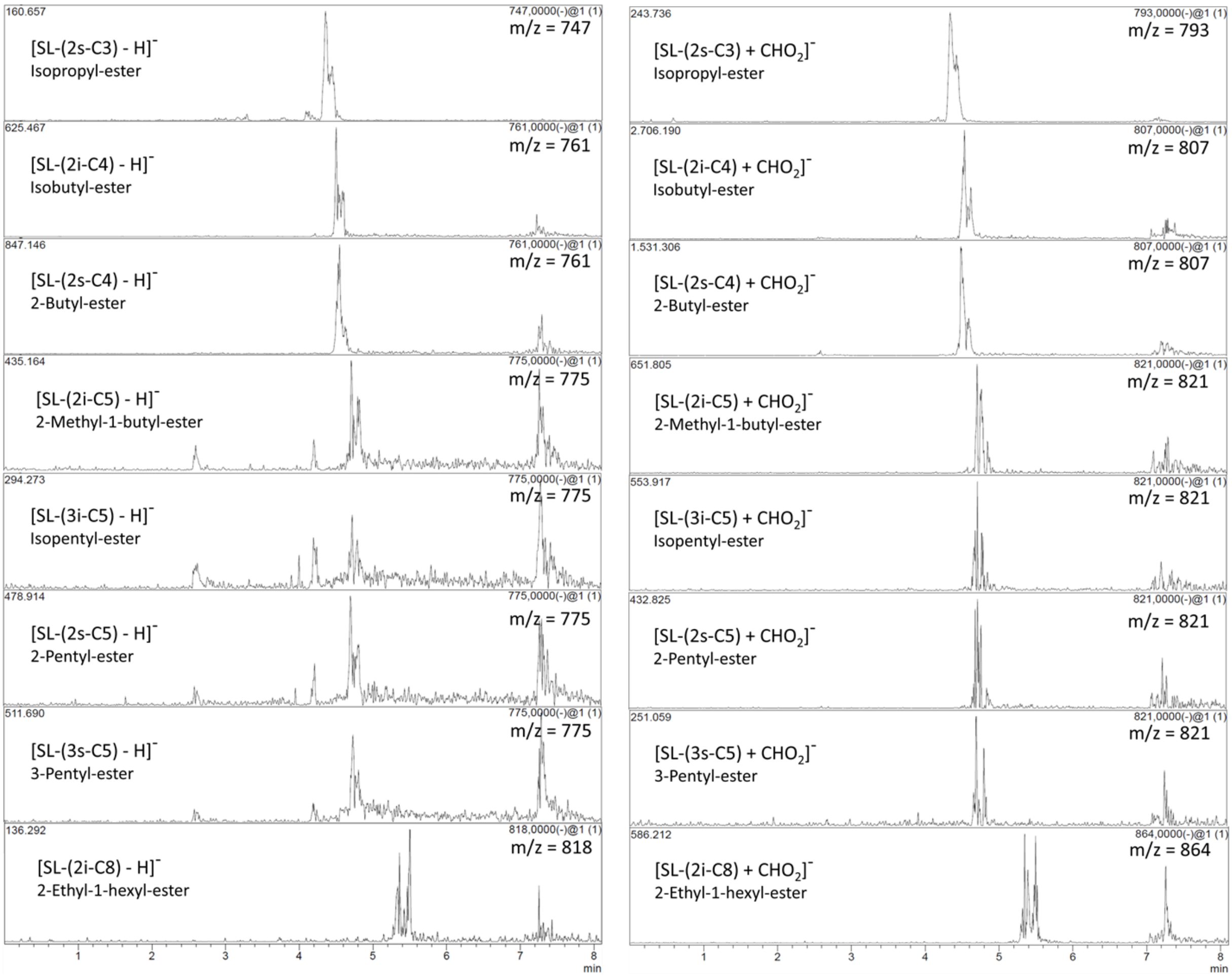

Fig S8 Overlay of ion chromatograms (EICs) for SL ester $[M - H]^-$ ions (left) and $[M + CHO_2]^-$ ions (right) of samples from cultivations of secondary and branched alcohols (m/z values for diacetylated SL species).

Table S3 Effect of alcohol feeding rate on total SL titer and ester fraction from *S. bombicola* cultivations supplemented with methanol, ethanol isopropanol and isobutanol at different dosage rates. The total SL titer is defined as the sum of lactonic, open-chain, bolaform SL and ester SLs.

Alcohol	Alcohol Feed [mM d^{-1}]	SL Titer [g l^{-1}]	SL Ester [%]	Bolaform SL [g l^{-1}]	Acidic SL [g/L]	Lactonic SL [g l^{-1}]	SL Ester [g l^{-1}]
Methanol	75	109 ± 12	8%	31 ± 10	5 ± 0	66 ± 5	9 ± 2
	200	102 ± 3	38%	14 ± 3	5 ± 1	45 ± 5	39 ± 6
	400	58 ± 9	44%	7 ± 2	4 ± 2	21 ± 5	25 ± 6
	800	40 ± 7	52%	3 ± 1	4 ± 1	13 ± 3	21 ± 7
Ethanol	75	71 ± 6	13%	6 ± 4	5 ± 1	51 ± 4	9 ± 1
	150	76 ± 7	44%	5 ± 1	6 ± 2	32 ± 3	33 ± 10
	250	71 ± 2	43%	5 ± 1	12 ± 2	24 ± 8	31 ± 10
	500	62 ± 7	47%	3 ± 2	19 ± 6	11 ± 3	29 ± 8
Isopropanol	100	68 ± 4	23%	4 ± 1	17 ± 9	32 ± 2	15 ± 3
	200	48 ± 2	35%	4 ± 1	9 ± 1	18 ± 3	17 ± 2
	300	27 ± 0	36%	0 ± 0	6 ± 0	12 ± 2	10 ± 2
	600	12 ± 3	34%	0 ± 0	2 ± 2	6 ± 2	4 ± 1
Isobutanol	50	94 ± 24	32%	4 ± 1	11 ± 6	49 ± 18	30 ± 13
	75	42 ± 13	45%	5 ± 1	5 ± 0	12 ± 3	19 ± 5
	100	31 ± 18	40%	0 ± 0	6 ± 1	13 ± 3	13 ± 1
	125	17 ± 9	29%	0 ± 0	2 ± 1	10 ± 6	5 ± 2

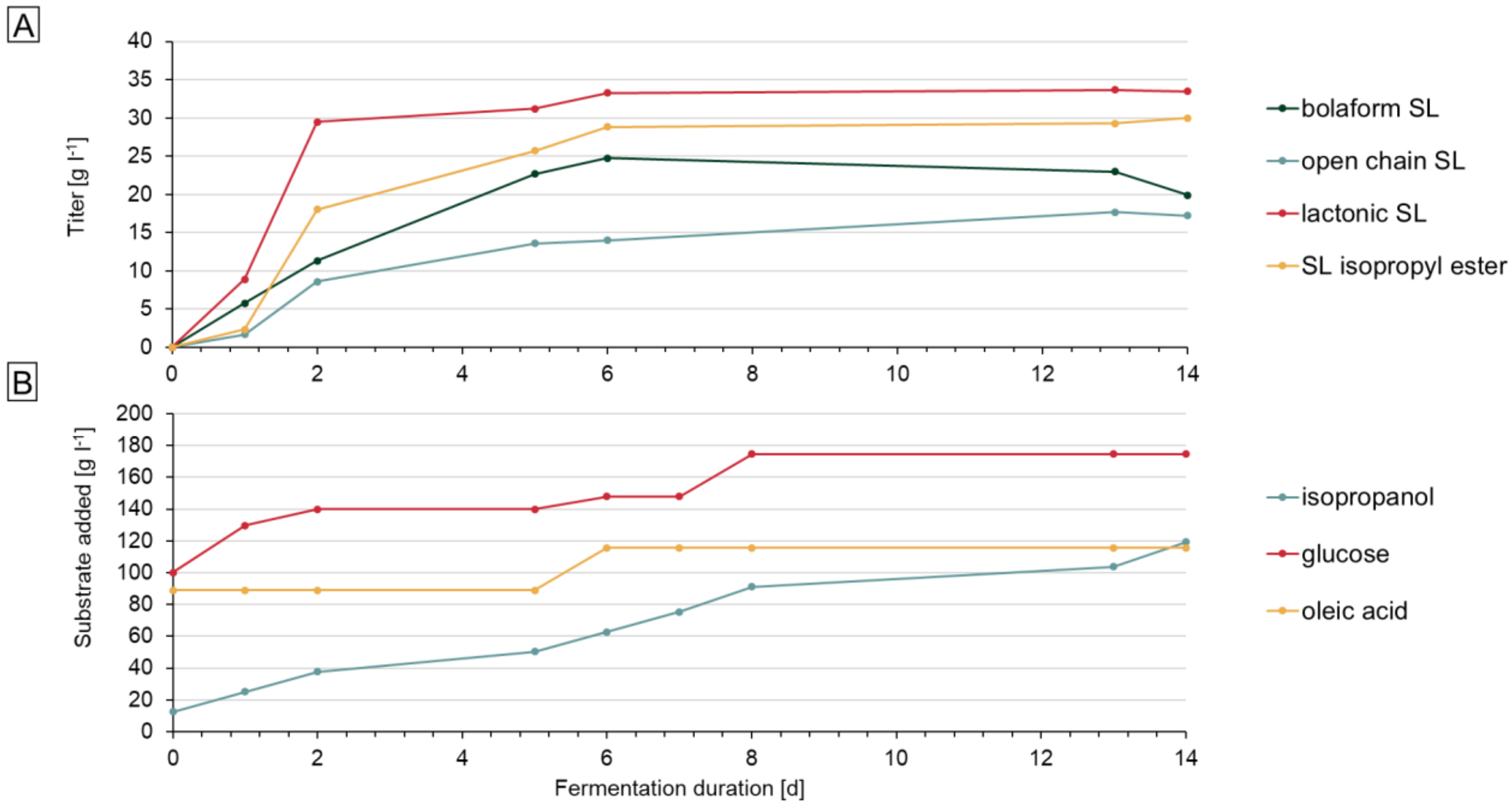

Figure S9 SL isopropyl ester production in a 2 l fed-batch fermentation with (A) formation of individual SL species and (B) cumulative amounts of substrate added over 14 days of fermentation.

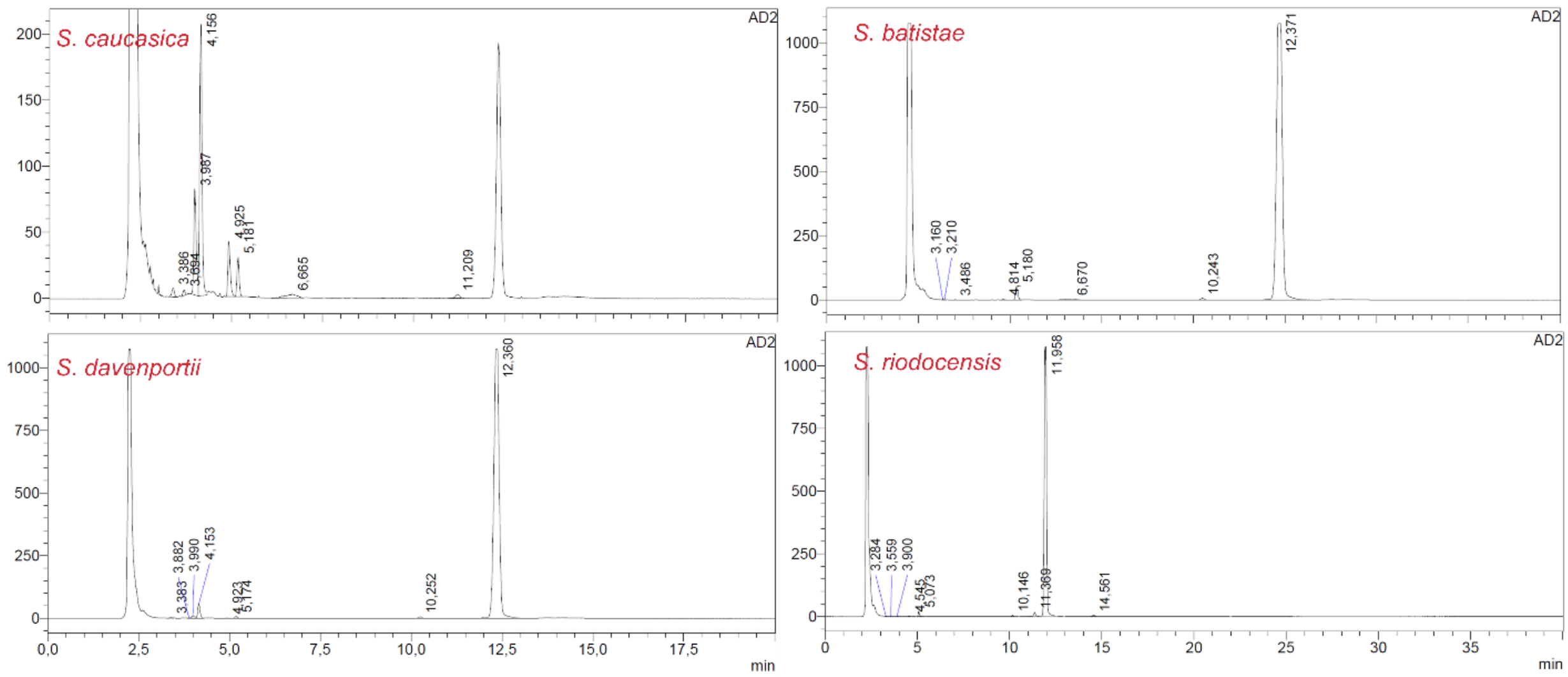

Fig S10 HPLC-ELSD chromatograms from cultivations of *S. caucasica*, *S. davenportii*, *S. batistae* and *S. riodocensis*. Signals at Rt = 3.99 min and Rt = 4.15 are tentatively assigned to non-acetylated open chain SLs (ω-1 and ω respectively). Signals at 5.07 min and 5.18 min belong to ω-1 and ω- diacetylated open chain SL and early eluting signals at 3.39 min belong to diacetylated bolaform SL. The signal at 4.92 min (*S. caucasica* and *S. davenportii*), 4.81 min (*S. batistae*) and 5.54 min, 11.37 min and 14.56 min (*S. riodocensis*) could not be attributed to a known congener.

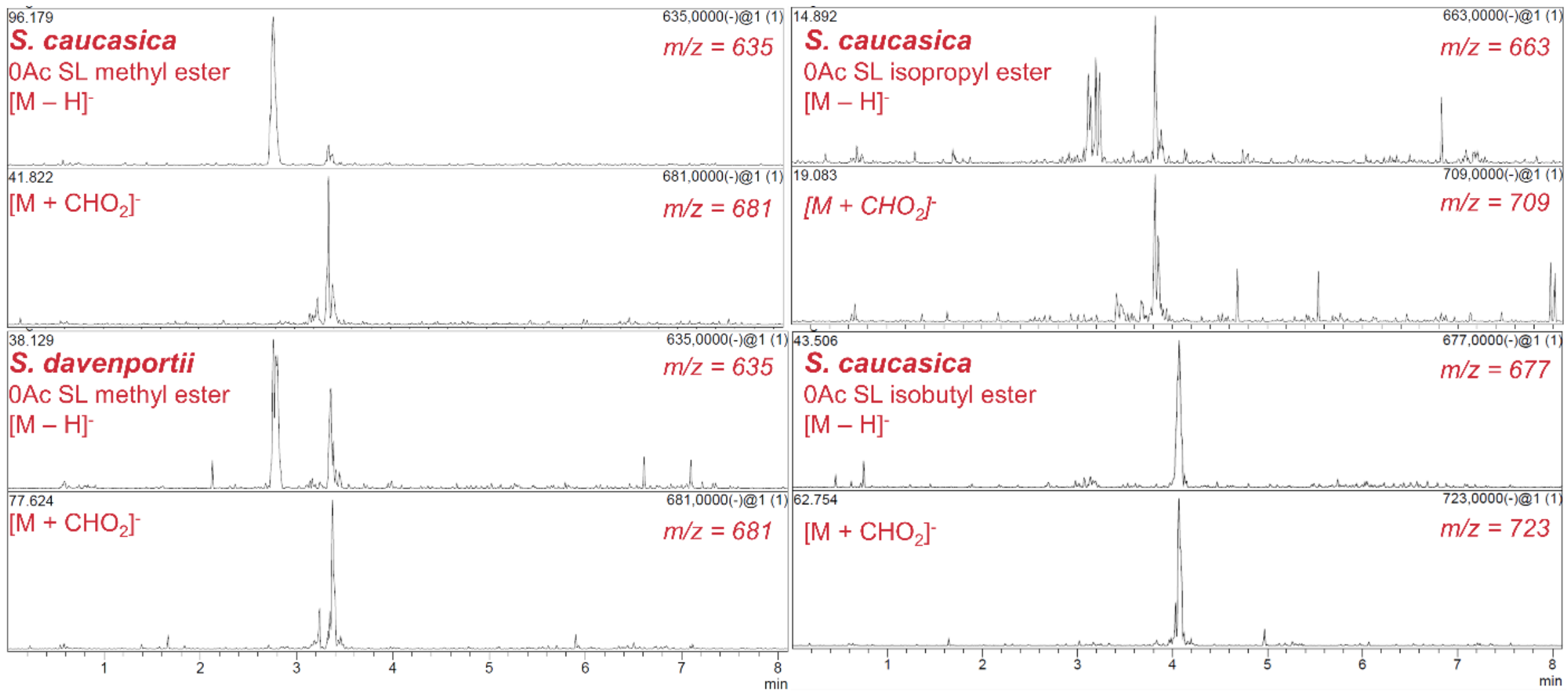

Figure S11 Extracted ion chromatograms of non-acetylated SL ester [M – H]- and [M + CHO_2]$^-$ molecule ions after feeding methanol to *S. davenportii* and methanol, isopropanol or isobutanol to *S. caucasica*.

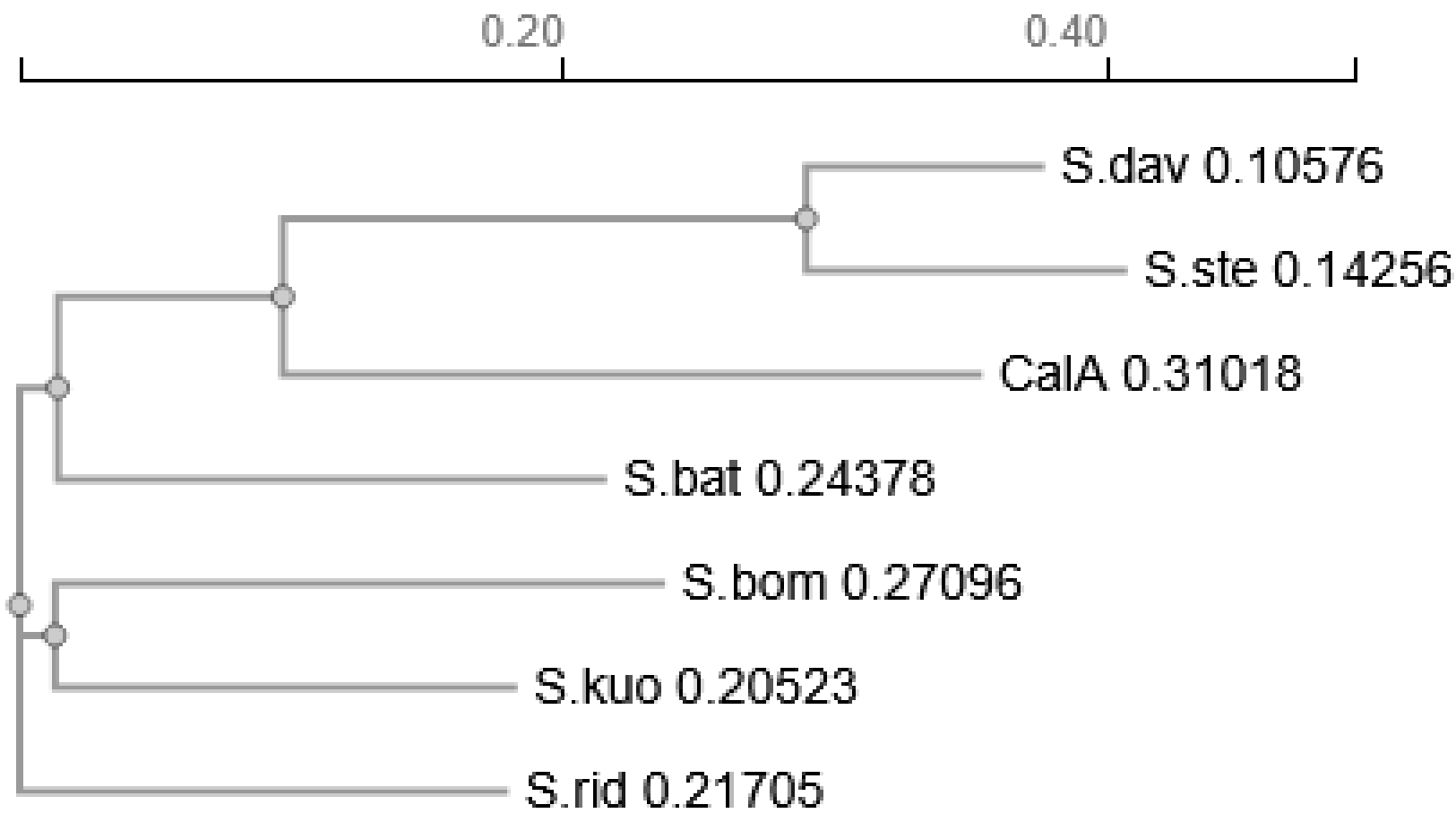

Figure S12 Phylogenetic analysis of sequence homologs to *Starmerella bombicola* lactone transesterase SBLE (S.bom) based on Clustal Omega multiple sequence alignment with sequences from S.dav = *Starmerella davenportii*, S.ste = *Starmerella stellata*, S.bat = *Starmerella batistae*, S.kuo = *Starmerella kuoi*, S.rid = *Starmerella riodocensis* and CalA = lipase A from *Pseudozyma aphidis* (formerly *Candida antarctica*)

Table S4 A) Analysis of sequence identities to *S. bombicola* SBLE using the EMBOSS NEEDLE pairwise sequence alignment tool and B) percent identity matrix from Clustal Omega multiple sequence alignment with the translated protein sequences shown below.

**A)**	**Identity**	**Similarity**	**Gaps**	**Score**
S. kuoi	199/397 (50.1%)	264/397 (66.5%)	19/397 (4.8%)	1026.0
S. batistae	165/396 (41.7%)	248/396 (62.6%)	25/396 (6.3%)	878.0
S. riodocensis	182/397 (45.8%)	245/397 (61.7%)	22/397 (5.5%)	930.0
S. davenportii	45/402 (11.2%)	74/402 (18.4%)	260/402 (64.7%)	196.5
S. stellata	49/403 (12.2%)	83/403 (20.6%)	245/403 (60.8%)	198.5
Lipase CalA	131/483 (27.1%)	189/483 (39.1%)	130/483 (26.9%)	473.5

**B)**	S.dav	S.ste	CalA	S.bom	S.bat	S.kuo	S.rid
S.dav	100.00	75.17	35.86	30.34	29.66	29.45	32.88
S.ste	75.17	100.00	30.86	26.54	28.40	27.61	26.38
CalA	35.86	30.86	100.00	30.15	35.60	33.33	34.95
S.bom	30.34	26.54	30.15	100.00	44.47	52.38	48.53
S.bat	29.66	28.40	35.60	44.47	100.00	51.61	53.23
S.kuo	29.45	27.61	33.33	52.38	51.61	100.00	57.29
S.rid	32.88	26.38	34.95	48.53	53.23	57.29	100.00

> *S. bombicola*
LPLGYTAAPAESFYFWPENISSLQAGEIFRKRELLTLPDIFDFGPNLEKVVQVAYKTRLTDGNDSFSIAS
IFIPKNPSPELKLYSYQTFEDAVQLDCAPSYALEVGNKSSNYLPVTSNLSAISRELEKGRHCIIPDHEGYI
SGFFAGRQEGYAGLDGIRAARNYLNGTNETPIGIFGYSGGAQATAWIVDLHDEYAPDLNFVGTVSGG
TLVDAWGTFQYIDYPKVYLKGSILIMYTGLFSGYPAQFEVIWPYIEPVIQENMLLLRLAPNDCNQSPILQ
GYNNSIMAGIHVDLPEFPASKYIFQHESLLANYSVVPVSTPKFPRYMYHGGSDELAKLSLVEQYVDQQ
WNTGANLTFVVYPGLLHDETAYRGFDAAMDWLDAQLDSGYLPPVNSTHT

> *S. kuoi*
GTGARPSDPFYDAPDNLGSYKNGEVFRRRSIPLTLDLGVKVSGVVQVAYKTQLADGGDSYSISTIFKP
ENPNSRIELFSYQIFEDAVQLDCAPSWTFKEGFFTPGVSVIDNNVTLAFSTQLEQGRYVVVPDHEGAV
SAFFCGRQEGYAGLDGIRAAINELNATNDNTPVIMYGYSGGAHATAWMVNVQDYYAPDLNVIAAAYG
GTLVDALGELNYADIPSDPVKGSVAAMYTGLLVGYPAQNETIWSHISQYIQNTILQLRWTPNLCSSQVL
LQNYNKSIRENIDVDIATFPAFVEIFKNESLLTSYSGQPVATPKYPRYIYHGTNDEIAPYNLTLSYINQQK
ASGGNIDWVVYNGLGHIEAGFAGENAAYDWIDQQFDN

> *S. riodocensis*
SVPNDPFYDVPPNLDQYKPGDIIRRREISQKFWVDANISKIVEVAYKTNLALGGDTWSIATIITPGNPDP
NLKLYSYQFFEDAVQLSCAPSWSLKNGLFSPGISSVVNSLTGTVGSQLRAGRHVVVPDHEGYISAFFS
GRQEGKAGLDGIRAALNELNASVSDTPVALYGYSGGGHATAWMVNLQPDYAPELNIVGAAYGGVLID
ALKELQWTDQKGAEAKGSVAALLTGLLLGNPPANESLWSHFSPYLKTKIYQLRTIPNFCSSELLWLDS
DDTIQSNIDVDIATFPPAVQMFKKETLLSNYSSIPIPTPKFPRYIYQGVDDTLAPYYLVKQYVDQQLATG
ANIEFVPYPGLDHIPVAFKGLDAAMDWIDQVFDK

> *S. batistae*
AVPNDSFYDPPTNLSDYNYGDVIRRRSIQQKFWQKATIKEIIQVQYKTRLTSGEDSWSISTVLVPSNPG
AKKQLYSYQVFEDAVQLDCAPSWTIKNGILSPGFGGITNAVADSIEGQVDQGRYVVVPDHEGAESAFF
GGRQEGYAGLDGIRAAINAVDLPKDTGVVIFGYSGGAHATAWMVNQWESYAKDINLIGAAYGGTLVD
AYQQIVYDDHYESLVKGSVASLIAGLLVAYPDVNSSLWSHITPDIQTAIVQMRIQPGGCDSAAFLTNFQ
GTVRAGINVNLSSFEPTKRMFQQETLLANLSSIPIPAPKFPRYIYHGSADEVAPYELIKEYVDEQESKGA
NITFKTYDGYGHVQTAFNGLQDAENWINS

> *S. davenportii*
IDKAFQMVYSSQDNYGGATSSVADVLIPRKDKLNGNIVAYQIAEDAVNIDCSPSYNLLYKRAIPAIQSLL
NEGYVVVVPDYEGVRGSFTVGKVAGRQILDALAATKERVSQLGLYQGDINVGVWGYGTAGWASGW
AAEIAEEYFPELKL

> *S. stellata*
IETAFQMVFSTQDNYERPSSSVADVLVPLKSKVNGNIVAFQIEQDSVNIDCVPSYNLFFKRTIPAIQSLL
NEGYVVVVPDYEGVRNSFSVGKIAGRSILDALSATKERVEQMKLFNGDLKVGVWGYGTAAWASGWA
AELAEEYSPDLHLKVAALGNYITNLTNWAT

> Lipase CalA
APATETLDRRAALPNPYDDPFYTTPSNIGTFAKGQVIQSRKVPTDIGNANNAASFQLQYRTTNTQNEA
VADVATVWIPAKPASPPKIFSYQVYEDATALDCAPSYSYLTGLDQPNKVTAVLDTPIIIGWALQQGYYV
VSSDHEGFKAAFIAGYEEGMAILDGIRALKNYQNLPSDSKVALEGYSGGAHATVWATSLADSYAPELN
IVGASHGGTPVSAKDTFTFLNGGPFAGFALAGVSGLSLAHPDMESFIEARLNAKGQQTLKQIRGRGFC
LPQVVLTYPFLNVFSLVNDTNLLNEAPIAGILKQETVVQAEASYTVSVPKFPRFIWHAIPDEIVPYQPAA
TYVKEQCAKGANINFSPYPIAEHLTAEIFGLVPSLWFIKQAFDGTTPKVICGTPIPAIAGITTPSADQVLG
SDLANQLRSLNGKQSAFGKPFGPITPP
